# An antibacterial compound pyrimidomycin produced by *Streptomyces* sp. PSAA01 isolated from soil of Eastern Himalayan foothill

**DOI:** 10.1038/s41598-022-14549-4

**Published:** 2022-06-17

**Authors:** Prasenjit Das, Shampa Kundu, Pulak Kumar Maiti, Saurodeep Mandal, Prithidipa Sahoo, Sukhendu Mandal

**Affiliations:** 1 Laboratory of Molecular Bacteriology, Department of Microbiology, University of Calcutta, 35, Ballygunge Circular Road, Kolkata, 700019 India; 2 The Molecular Recognition Laboratory, Department of Chemistry, Visva-Bharati University, Siksha Bhavana, Santiniketan, Birbhum, West Bengal 731235 India

**Keywords:** Antibiotics, Antimicrobial resistance, Antimicrobials, Applied microbiology

## Abstract

Selective isolation of soil Actinobacteria was undertaken to isolate a new class of antibiotics and bioactive molecules. A *Streptomyces* sp*.* PSAA01 (= MTCC 13,157), isolated from soil of Eastern Himalaya foothill was cultivated on a large scale for the production of the antimicrobial SM02. It has been found that the maximum amount of SM02 produced while PSAA01 was grown in ISP-2 medium (pH 7.0) for 7 days at 30 °C in shaking (180 rpm) condition. A significant zone of inhibition against *Staphylococcus aureus* MTCC 96 has been found with the crude cell-free culture media (50 µL) of 7 days grown PSAA01. After the purification and chemical structural characterization, we found that SM02 is a new antimicrobial having 746 dalton molecular weight. The compound SM02 contains pyrimidine moiety in it and is produced by a species of *Streptomyces* and thus we have named this antibiotic pyrimidomycin*.* The antimicrobial spectrum of pyrimidomycin has been found to be restricted in Gram-positive organisms with a MIC of 12 µg/mL. SM02 was found active against *Mycobacterium* sp. and also multi-drug resistant Gram-positive bacteria with similar potency and found to disrupt the bacterial cell wall. Pyrimidomycin also showed significant impairment in the biofilm formation by *S. aureus*. Furthermore, pyrimidomycin showed synergy with the most used antibiotic like ampicillin, vancomycin and chloramphenicol. Pyrimidomycin did not have cytotoxicity towards human cell lines indicating its limited activity within bacteria.

## Introduction

Actinomycetes are known to be the most common source of antibiotics. *Streptomyces* is a very common genus for antibiotic production^1^. A diverse group of antibiotics derived from actinomycetes is used broadly in medicinal purposes, for example, β-lactams, tetracyclines, macrolides, aminoglycosides, or glycopeptides^2^. Soil has been regarded as a great reservoir of actinobacterial strains^3,4^. It has been estimated that around 45% of the bioactive compounds and 80% of antibiotics obtained from actinomycetes are derived from the two genera, *Streptomyces* and *Micromonospora*^5,6^. Actinobacteria generally produce antibiotics when they are exposed to diverse stress conditions. Stress conditions might often appear through nutrient deprivation which induces actinobacteria, especially *Streptomyces* to transform from vegetative mycelial structure to erected sporogenic aerial mycelia^7^. In addition to the nutrient deprivation, the presence of different neighbouring genera is also a limiting factor for the actinobacterial growth and it also induces antibiotic production to make its growth smooth.

*Streptomyces* are in general filamentous, Gram-positive bacteria present in all types of environments with a high G+C content in their genome. It has been estimated that soil itself contains about 90% of total actinobacteria. Due to the presence of diverse *Streptomyces* species on a large scale, the isolation of novel antibiotic-producing species should be very specific^8^.

Recently, the treatment of most infectious diseases and control of different pathogenic organisms have become one of the key challenges as the uses of the antibiotics are compromising which makes the pathogens turn resistant against most of the antibiotics and making them multi drug-resistant pathogens (MDR pathogens)^9,10^. There are several strategies to overcome the antibiosis by antimicrobial agents such as enzymatic inactivation, target modification, influx impairment and excessive efflux are being followed by most of the MDR pathogens^10,11^. The most common strategy of the MDR pathogens to be resistant against the antimicrobial agent is biofilm formation^12^. The problem of the exponential rise of antimicrobial resistance can be mitigated with discovery of new antimicrobial agents produced by other microbes, as the majority of clinically relevant antibacterial agents (75%) are natural products of the microbial origin or their analogs^10,11,13^.

There are many types of antibiotics which are having different targets for the inhibitory effects on the microorganisms. Formation of DNA, cell wall, proteins are the major targets for most of the antibiotics to inhibit the organisms. Beta-lactam drugs like, carbenicillin, penicillin G, cefuroxime, aztreonam, cefoperazone, oxacillin, ampicillin and a glycopeptide drug, vancomycin target the cell wall synthesis of the organisms and inhibit them^14,15^. On the other hand, oleandomycin, clindamycin, lincomycin, tobramycin, chloramphenicol, gentamycin, streptomycin, etc. block cellular protein synthesis to inhibit the microorganisms^16–19^. Besides these, another category of antibiotics which includes levofloxacin, novobiocin, rifampicin, ofloxacin, nitrofurantoin, etc. target nucleic acid synthesis^20^. However, some antibiotics may affect even the eukaryotic systems by interfering with several cellular functions^21^ and thus it is very crucial to check the toxic effect on human cells.

In this study, an antimicrobial producing *Streptomyces* sp. PSAA01 has been isolated along with 25 other actinobacteria from the soil sample collected from Manas National Park, Assam, India. The antimicrobial production has been optimized taking various media, culture time and media pH. The chemical structural elucidation of the antibiotic molecule has also been revealed using CHNS/O analysis, mass spectrometry, FTIR, ^1^H NMR, ^13^C NMR, UV–Visible and fluorescence spectroscopy. The antimicrobial and antibiofilm properties of the molecule have been investigated using multiple test pathogens. The cytotoxicity of the molecule has further been checked against the human cell line and probable mode of action has been deciphered.

## Methods

### Selective isolation of actinomycetes

The soil samples were collected from Manas National Park, Chirang and Baksa (26° 65′ N-91 E; altitude: 200 ft; annual rainfall: 333 cm and a temperature range from 15 to 37 °C). In order to enrich and selectively isolate the actinobacteria, the soil samples were subjected to pre-treatment with CaCO_3_ and incubated for 7 days at room temperature followed by incubation for 2 h at 65 °C in a hot air oven^22,23^. 1 g of the pre-treated soil was dissolved in 1 mL of 0.9% NaCl and diluted sequentially up to 10^−6^. Each 0.1 mL of the diluted sample was spread on a starch casein medium. The medium was supplemented with cycloheximide (50 μg/mL) and nystatin (50 μg/mL) to inhibit undesired fungal growth. After incubation for 3–4 days at 30 °C, the inoculated plates showed different actinobacterial colonies (25). Each of the colonies having different and unique features has been selected and streaked on fresh ISP-2 (International Streptomyces Project) plate^22,25^.

### Identification of the PSAA01

An isolate, PSAA01 with differential mycelia (aerial and substrate mycelium) and diffusible pigments, was incubated for 14 days on ISP-2 medium^25^. Various colony morphology like colony size, shape, texture, margin, optical properties was observed on 14 days of incubation on ISP-2 medium. The cellular morphology has been observed under Scanning Electron Microscope following the standard protocol^26^. The 16S rDNA was amplified with the help of 8F and 1492R universal primers followed by sequencing of the amplicon and further sequence analysis using BLASTn program (https://blast.ncbi.nlm.nih.gov) and EZbiocloud server^27^. 16S rDNA sequences of the closest type strains were obtained from the EZbiocloud server^27^ and the phylogenetic analyses were performed using Neighbour Joining (NJ)^28^ and Maximum Likelihood (ML)^29^ algorithms. To reveal the evolutionary status of PSAA01, the distance-based and character-based phylogenetic trees were constructed. The 16S rDNA sequence of the strain was deposited in NCBI and the accession number is MT829328.

### Preliminary screening for antimicrobial property

Antimicrobial activity of the strain PSAA01 was examined in Mueller–Hinton (MH) agar media following the standard agar-diffusion method^30^. In this method, 5–7 days old colony of PSAA01 (10 mm in diameter) was picked and inoculated on the plate which is just spread with exponentially grown test organisms such as *Staphylococcus aureus* MTCC 96, *Bacillus cereus* MTCC 1272 (Gram-positive), *Escherichia coli* MTCC 1687, and *Pseudomonas aeruginosa* MTCC 424 (Gram-negative). For a homogenous distribution of antimicrobial compound, already produced in the inoculum, the plates were kept at 4 °C for 2 h followed by the incubation at 37 °C for further 24 h^26^.

### Culture conditions for SM02 production

The optimal production of the antimicrobial compound produced by PSAA01 has been explored with various media conditions, incubation time and the media pH. Five media AIA^25^, ISP-2^31^, ISP-3^31^, tryptic soya broth (TSB)^32^, and starch casein (composition: soluble starch—10 g, K_2_HPO_4_—2 g, KNO_3_—2 g, casein—0.3 g, MgSO_4_.7H_2_O—0.05 g, CaCO_3_—0.02 g, FeSO_4_.7H_2_O—0.01 g, agar—15 g, water—1000 mL and pH—7.0 ± 0.1) were selected by virtue of the differences in their composition^24^ while growth parameters like temperature (30 °C), pH (8), shaking speed (180 rpm), time (7 days), and inoculum (1%) were kept constant. The medium which showed the maximum antibiotic production (derived comparing the efficacy of its antibiosis) was selected for the all-downstream experiments. The timepoint and pH for the maximum antibiotic production in the selected medium were also investigated. The medium which allows the maximum production of antibiotics was checked by measuring the wet weight of the cell mass of the strain PSAA01 inoculated in five different media.

### Preliminary screening for spectrum of activity of crude SM02

The antibiosis efficacy of the crude SM02 produced by the isolate PSAA01 was determined against 6 different Gram-positive test organisms (*S. aureus* MTCC 96, *Streptococcus pyogenes* MTCC 1928, *B. cereus* MTCC 1272, *B. subtilis* MTCC 441, Methicillin-resistant *S. aureus* (MRSA), *Mycobacterium smegmatis* mc^2^ 155) and 4 different Gram-negative test organisms (*E. coli* MTCC 1687, *P. aeruginosa* MTCC 424, *Klebsiella pneumoniae* MTCC 109, *Salmonella typhi* MTCC 733) and compared with the standard antibiotics like ampicillin (10 μg) and chloramphenicol (4 μg). To determine the antimicrobial efficacy of the compound, 0.1 mL culture of 0.1 OD (A_600_ nm) cell of test organisms were seeded on MH agar medium and the 50 μL of the cell-free extract was applied in the well made in each plate along with the standard antibiotic in independent well^26^. The plates were incubated for 24 h for all the test organisms and 48 h for *M. smegmatis* mc^2^155. After the incubation the zone of inhibition was observed.

### Production, extraction, and purification

The strain PSAA01 was inoculated in a freshly prepared sterile ISP-2 medium (volume: 3 L) and incubated at 30 °C for 7 days in shaking (180 rpm) condition. After the incubation, the medium was centrifuged for 15 min at 13,000 rpm to collect the supernatant. An equal volume of ethyl acetate with the supernatant was added to extract the active compounds. After extraction, the active organic phase was collected and dried by a rotary evaporator. The crude mixture was subjected to load on a preparative thin layer chromatography (TLC) plate (Silica gel 60 F_254_) using PET:CHCl_3_ (1:1,v/v), R_f_ = 0.30. The collected TLC-spot with desired activity was then run into flush column chromatography consisting of silica gel bed (60–120 mesh) with the same eluent ratio of the solvents to purify the compound. The pure product was obtained as light brown solid after the evaporation of the solvent from the eluted fractions through the rotary evaporator. We obtained 40 mg pure product from the 3L extract solutions. The purified product was then used further for its chemical and functional characterization.

### Estimation of MIC and MBC

To determine the minimum inhibitory concentration (MIC), 190 μL MH medium and 10 μL stock solutions (7.5, 15, 30, 60, 120, 240, 480, µg/mL) of SM02 compound derived from PSAA01 was mixed into the well of 96-well plate in order to get a final concentration of SM02 at 0.375, 0.75, 1.5, 3, 6, 12, and 24 µg/mL. 5 × 10^5^ cells were used for the inoculation into each experimental set of both MIC and minimum bactericidal concentrations (MBC) assay. After adding SM02, the 96-well plate was incubated at 37 °C for 24 h at shaking conditions (180 rpm). MIC was recorded as the lowest SM02 concentration where visible growths of the test organism were absent. Minimum bactericidal concentrations (MBC) were analyzed in microplates through the broth dilution method^33^. 45 μL sterile MH broth along with 5 μL (240, 480, 960 and 1920 µg/mL) SM02 compound and 50 μL test inoculum was mixed and volume adjusted to 100 μL to get a final concentration of SM02 at 12, 24, 48 and 96 µg/mL, followed by the incubation at 37 °C for 24 h shaking condition (180 rpm). After incubation, 10 μL cultures from each well were taken for colony counts and were spread on Luria Bertani agar plates and incubated at 37 °C for 24 h. MBC was noted as a culture with a minimum concentration of the compound SM02 in which no colony was found on the agar plate.

### Observation under scanning electron microscopy

To investigate the alteration of the cellular morphology of the test organisms *B. cereus* MTCC 1272 and *S. aureus* MTCC 96 after treatment with the compound SM02. Scanning electron microscopy (ZEISS-EVO-MA-10) was performed according to the standard protocol^34^. The test organism was treated with SM02 compound (12 μg/mL) in MH medium^30^, followed by incubation at 37 °C for 24 h in shaking condition (180 rpm). The treated samples were subjected to centrifugation and fixed by 2.5% glutaraldehyde solution in phosphate buffer for 30 min. The fixed cells were again centrifuged and washed thrice with 0.1 M phosphate buffer (pH 7.2). Then each suspension was serially dehydrated with 25%, 50%, 75%, 90%, and 100% ethanol. The samples were placed under the microscope and images were captured.

### Antibiofilm assay

The antibiofilm property of SM02 was determined using the crystal violet assay. 100 μL of *S. aureus* MTCC 96 (O.D was adjusted to 0.5 according to McFarland protocol) was treated with 5 μL SM02 compound of sub-MIC concentrations (1.5 µg/mL, 3 µg/mL and 6 µg/mL) in MH broth and incubated at 37 °C for 24 h in shaking condition. After incubation, planktonic cells were discarded and washed with sterile water. Then 100 μL of 0.1% crystal violet solution was added and incubated for 10–15 min at room temperature. The excess crystal violet was washed 3–4 times with sterile water and dried for a few hours. The O.D was documented at 550 nm in a 96-well plate reader after diluting these ten-fold^35^.

### Cytotoxicity assay

HuH-7 cell lines (RRID: CVCL_0336) were prepared from the continuous culture in Dulbecco’s Modified Eagle’s Medium (DMEM, Sigma Chemical Co., St. Louis, MO) supplemented with 10% fetal bovine serum (Invitrogen), penicillin (100 μg/mL), and streptomycin (100 μg/mL). Cells were initially propagated in a 75 cm^2^ polystyrene, filter–capped tissue culture flask in an atmosphere of 5% CO_2_ and 95% air at 37 °C in a CO_2_ incubator. When the cells reached the logarithmic phase, the cell density was adjusted to 1.0 × 10^5^ per/well in culture media. The cells were then used to inoculate in a glass-bottom dish, with 1.0 mL (1.0 × 10^4^ cells) of cell suspension in each dish. After cell adhesion, the culture medium was removed. The cell layer was rinsed twice with phosphate-buffered saline (PBS) (pH 7.0). Cells were centrifuged at 1200 rpm for 10 min after treatment with trypsin. The supernatant has been discarded and the fresh medium was added to the pellet to adjust 10^4^ cells in 100 μL medium followed by incubation overnight. All tests have been repeated atleast three times. Overnight grown cells were treated with different concentrations of SM02 compound and further incubated overnight. Upon incubation 20 μL MTT reagents (5 mg/mL) were added in each set and incubated at shaking condition for 4 h. The supernatants were discarded and 150 μL DMSO was added to dissolve formazan, formed due to the reaction of the MTT dye and NAD(P)H-dependent cellular oxidoreductase present only in viable cells. The absorbances were then measured at A_590_ nm^36^.

### Antimicrobial efficacy of pyrimidomycin in combination with other antibiotics

To determine the combinatorial effect of pyrimidomycin with the most common antibiotics used in the present treatment regimen, on bacterial pathogens, we have used the preliminary checkerboard method according to the standard protocol^37^. The concentrations of all antibiotics used for the experiment were MIC to 1/4 MIC. The final concentrations of antibiotics were: ampicillin 0.1, 0.2, 0.4 μg/mL; vancomycin 0.25, 0.5, 1 μg/mL; chloramphenicol 1, 2, 4 μg/mL; and pyrimidomycin 3, 6, 12 μg/mL. This test was performed in triplicate on 96 well plate taking 100 μL MH broth in each well. Each well contains 5 μL of first test compound (SM02), 5 μL of second test compound (another antibiotic), 50 μL of freshly diluted test organism and 40 μL of MH broth. The final inoculum was 5 × 10^5^ CFU/mL. Also, each well contain only the respective individual antibiotics along with inoculum of the test pathogen *S. aureus* MTCC 96. The cells were incubated in shaking conditions (180 rpm) for 16 h at 37 °C. After that OD was taken at 600 nm. In vitro interaction was measured as the fractional inhibitory concentration index (FICI), the formula of FICI calculation is (MIC of drug A in combination/MIC of drug A alone) + (MIC of drug B in combination/MIC of drug B alone). FICIs were interpreted as follows: < 0.5, synergy; 0.5–0.75, partial synergy; 0.76–1.0, additive effect; 1.0–4.0, indifference; and > 4.0, antagonism. The varying levels of synergy between two given agents were determined^37^. All experiments were repeated three times.

### Analytical experiments to characterize SM02

The solvents used were distilled and dehydrated according to the standard procedures^38^. The high-resolution mass spectrometry (HRMS) was performed using a micromass Q-TOF MicroTM instrument by using methanol as a solvent. ^1^H-, ^13^C- NMR spectra were collected at 400 and 100 MHz, respectively, on a Bruker DRX spectrometer. For NMR spectra, CDCl_3_ was used as solvent using TMS as an internal standard. Chemical shifts are expressed in δ ppm units and ^1^H–^1^H and ^1^H–^13^C coupling constants in Hz. The following abbreviations are used to describe spin multiplicities in ^1^H NMR spectra: s = singlet; d = doublet; t = triplet; m = multiplet. KBr pellets have been used to record the FTIR spectra using a spectrophotometer. Fluorescence spectra of the compound SM02 were recorded on a Perkin Elmer Model LS 55 spectrophotometer whereas the UV–Vis spectra were recorded on a SHIMADZU UV-3101PC spectrophotometer. Elemental analysis of the SM02 compound was carried out on CHNS/O analyzer. Specific rotation of the compound SM02 were recorded on Bellinham Stanley Ltd. polarimeter.

### Ethical approval

The research is not associated with any prior ethical approval.

### Consent of publication

The data used for the manuscript is original and does not require any consent from third party for publication.

## Results

### Isolation and identification of PSAA01

**Figure 1:**
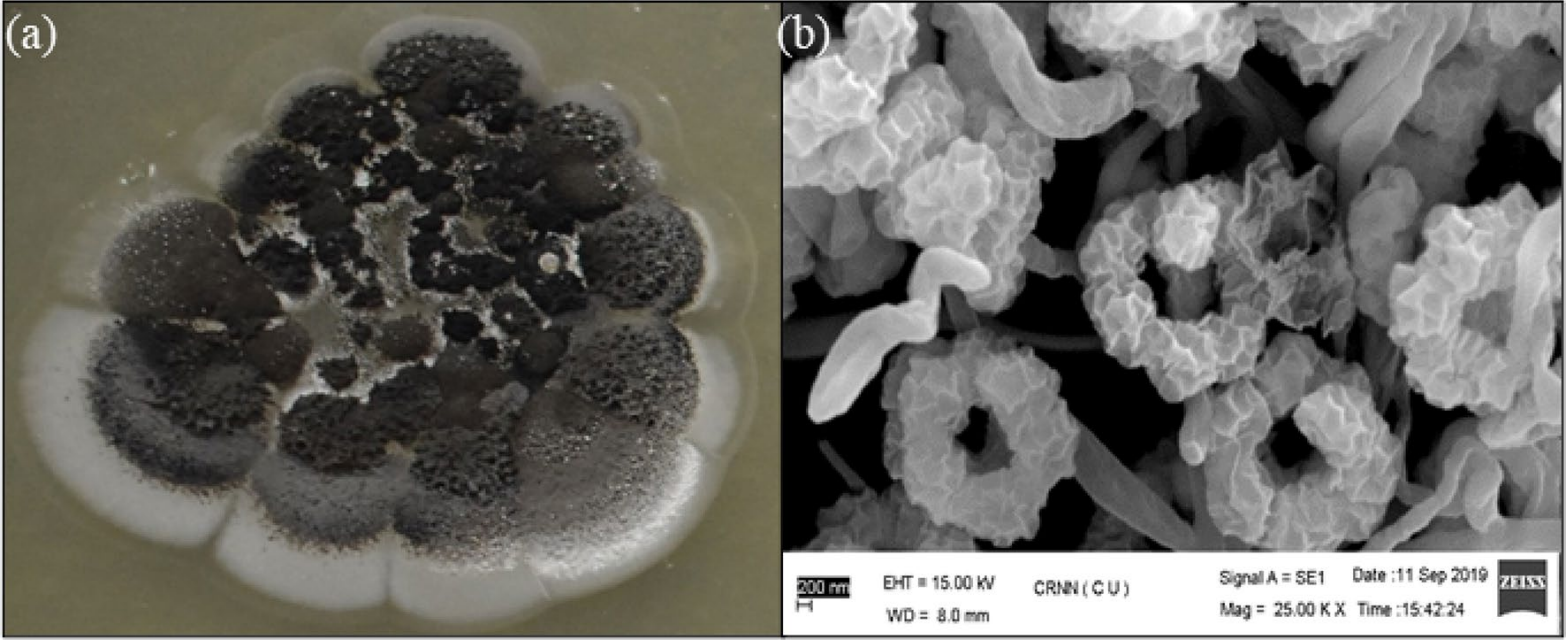
Morphology of the isolate PSAA01. (**a**) The colony morphology of the strain PSAA01; (**b**) the cell morphology of the filamentous actinomycete strain PSAA01 as observed under scanning electron microscopy in 25000X magnification.

A *Streptomyces* sp. PSAA01 (= MTCC 13157) was isolated from soil collected from Manas National Park, Assam, India. *Streptomyces* is the most prevalent actinomycetes present in the soil and have a huge contribution towards the development of pharmaceuticals. The strain was grown on the ISP-2 medium. The colony of the isolated strain has been found to be irregular, blackish and rough (Fig. 1a). Scanning electron micrograph of PSAA01 shows that the filamentous mycelia and the spore surfaces are rough (Fig. 1b). The 16S rDNA sequence (MT829328) of the strain PSAA01 (= MTCC 13157) is having a 99.72% similarity with *Streptomyces melanosporofaciens* DSM 40138, as analysed with EZBioCloud. Phylogenetic analysis with Neighbour Joining (NJ) algorithm shows that the isolate originated from the same ancestor as *S. melanosporofaciens* DSM 40138, but based on the branch length and other molecular and biochemical observation (data not shown), it can be assumed that the isolate might be quite different from its closest neighbour (Fig. S1). It has been found to secrete a bioactive metabolite SM02, which is having antimicrobial property.

### Culture conditions for SM02 production

ISP-2 medium was found to be the best-tested medium as the cell-free extract that contains the desired antimicrobials showed the highest inhibitory effect against *S. aureus* MTCC 96 (Fig. S2). To estimate the culture incubation time-point that has highest antimicrobial production, we prepared the cell-free extract and checked the antimicrobial potency. The maximum antibiosis on the test pathogen was assumed as the point where there is maximum SM02 production has occurred. The incubation has been continued for 15 days to get the interval time points. The production (as determined by the test-pathogen killing assay) was found to be maximum after 7 days of incubation (Fig. S3a). Similarly, it has been found that the optimum pH was 7.0 for SM02 production (Table S1; Fig. S3b). This indicates that the maximum efficacy of the desired compound SM02 can be obtained while the strain PSAA01 were grown in ISP-2 medium (pH 7.0) in shaken condition (180 rpm) for 7 days at 30 °C.

### Antimicrobial activity of crude extracts the contains SM02

The zone of inhibition of the cell-free crude extracts that contains SM02 compound were tested on various test organisms by agar-diffusion methods. Among the tested organisms, 7 Gram-positive test organisms (*S. aureus* MTCC 96, *S. pyogenes* MTCC 1928, *B. cereus* MTCC 1272, *B. subtilis* MTCC 441, Methicillin-resistant *S. aureus* (MRSA), *M. smegmatis* mc^2^ 155) has been found to be sensitive against the extract having SM02 , whereas no zone of inhibitions were observed against any of the 4 Gram-negative (*E. coli* MTCC 1687, *P. aeruginosa* MTCC 424, *K. pneumoniae* MTCC 109*, S. typhi* MTCC 733) organisms, indicating that SM02 is active against Gram-positive bacteria (Fig. S2; Fig. S4).

### Structural elucidation of SM02 compound

**Figure 2:**
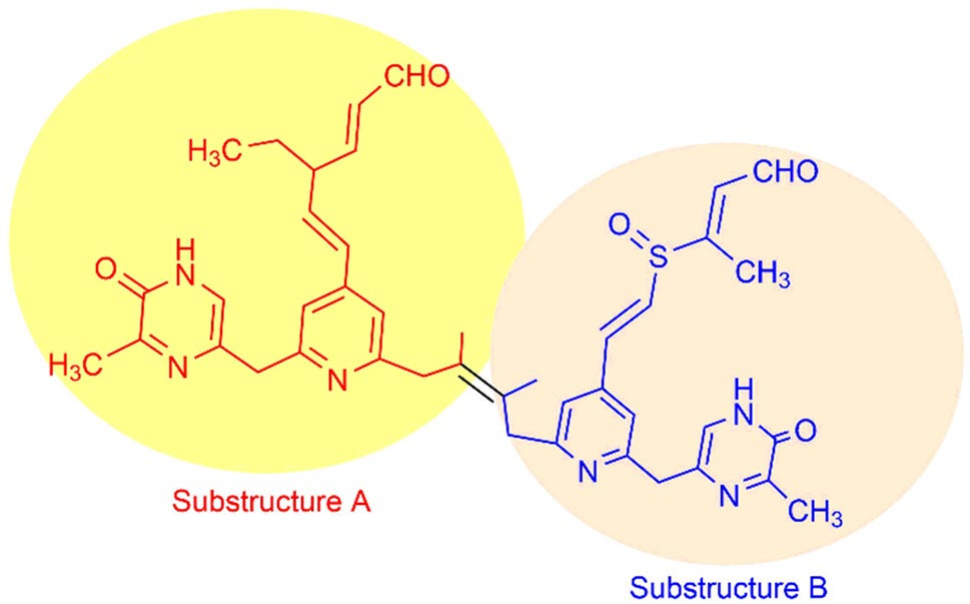
The total structure of pyrimidomycin with substructure A (in red) and B (in blue).

Structural characterization was performed by CHNO/S analysis, mass spectrometry, FTIR, ^1^H NMR, ^13^C NMR, UV–Visible and fluorescence spectroscopy. The mass spectrum of SM02 showed a base peak [M + H]^+^at m/z 747.4687 (Fig. S5) [calculated mass: 747.3329 (M + H^+^)] (Table 1). The molecular formula was determined to be C_42_H_46_N_6_O_5_S based on HRMS analysis data by considering the number of protons and carbons from NMR spectrum (Table 2). Sulfur atom is predicted to be present in SM02 as we performed special elements test by lassaigne test. IUPAC name of SM02 is (2E,5E)-6-(2-((Z)-2,3-dimethyl-4-(6-((6-methyl-5-oxo-4,5-dihydropyrazin-2-yl)methyl)-4-((E)-2-(((E)-4-oxobut-2-en-2-yl)sulfinyl)vinyl)pyridin-2-yl)but-2-en-1-yl)-6-((6-methyl-5-oxo-4,5-dihydropyrazin-2-yl)methyl)pyridin-4-yl)-4-ethylhexa-2,5-dienal (Fig. 2).

**Table 1:** Physicochemical properties of SM02.

Appearance	Gummy brown solid
Molecular formula	C_42_H_46_N_6_O_5_S
Molecular weight	746.9270
CHN analysis	C:67.53; H:6.23; N: 11.26; O:10.70; S:4.28
HRMS m/z	(M + H)^+^
Calcd	747.3329 (M + H^+^)
Found	747.4687
UV (Methanol)	280 nm
IR (KBr) cm^−1^ Optical rotation ([α]_D_^25ºC^	3354, 2934, 2832, 1710, 1661, 1456, 1380, 1027, 958, 744 ** + **60º (solvent used: chloroform)

**Table 2.** ^1^H and ^13^C NMR spectral data of SM02 in CDCl_3_.

Position	δ_H,_ J(Hz)	δ_c_	Position	δ_H,_ J(Hz)	δ_c_
1	9.61–9.63(d, J = 8)	200.34	23	–	122.03
2	7.01–7.05(t, J = 16)	145.48	24	1.59 (s)	14.44
3	5.75–5.77(t, J = 8)	111.94	25	3.13(s)	38.57
4	3.53–3.55 (t, J = 8)	51.82	26	–	170.28
5	2.52–2.55(q, J = 12)	29.81	27	–	170.28
6	0.96–0.98 (t, J = 8)	12.66	28	7.24 (s)	153.34
7	5.46–5.47(d, J = 4)	110.97	29	–	153.47
8	6.14–6.16 (d, J = 8)	126.38	30	7.24 (s)	153.34
9	–	153.47	31	6.32–6.35(d, J = 12)	130.53
10	7.24 (s)	153.34	32	6.63–6.66(d, J = 12)	133.57
11	–	170.28	33	–	143.43
12	–	170.28	34	7.10–7.12(d, J = 8)	146.18
13	7.24 (s)	153.34	35	9.61–9.63(d, J = 8)	209.05
14	3.13(s)	45.29	36	1.38(s)	21.16
15	–	165.53	37	3.13(s)	45.29
16	7.75(s)	156.49	38	–	165.53
17	–	182.54	39	7.75 (s)	156.49
18	–	166.13	40	–	182.54
19	2.03(s)	23.14	41	–	166.13
20	3.13(s)	38.57	42	2.03 (s)	23.14
21	–	122.03	NH^a^	7.21–7.22(d, J = 4)	
22	1.59(s)	14.44	NH^b^	7.21–7.22(d, J = 4)

The compound showed absorption maxima at 280 nm and emission maxima at 400 nm (Fig. S6, S7). Absorption at 3354, 2934, 2832, 1710, 1661, 1456, 1380, 1027, 928, 744 cm^−1^ in the FTIR spectrum suggests the presence of N–H stretching (Amide), C–H stretching (Alkane), C–H stretching (Aldehyde), C=O stretching (Aldehyde), C=O stretching (Amide), C–H bending (Alkane, methyl group), C–H bending (Aldehyde), S=O stretching (Sulfoxide), C=C bending (Alkene, trans disubstituted), C=C bending (Alkene, trisubstituted) (Table S2, Fig. S8). The detailed NMR studies (^1^H NMR, ^13^C NMR, DEPT-135, COSY, HMBC, HSQC and NOSEY) (Fig. S9-S16) were performed to establish the structure of SM02. ^13^C NMR spectrum (Fig. S10) showed 26 signals that were assigned to 6 methyl, 7 methine, and 15 quaternary carbons. DEPT-135 NMR spectrum (Fig. S11) showed 17 signals in which 14 positive signals and 3 negative signals that suggest the presence of 14 types of CH/CH_3_ carbons and 3 types of CH_2_ carbons in SM02. The total structure of SM02 consists of substructures A and B (Fig. 2).

### Substructure A

**Figure 3:**
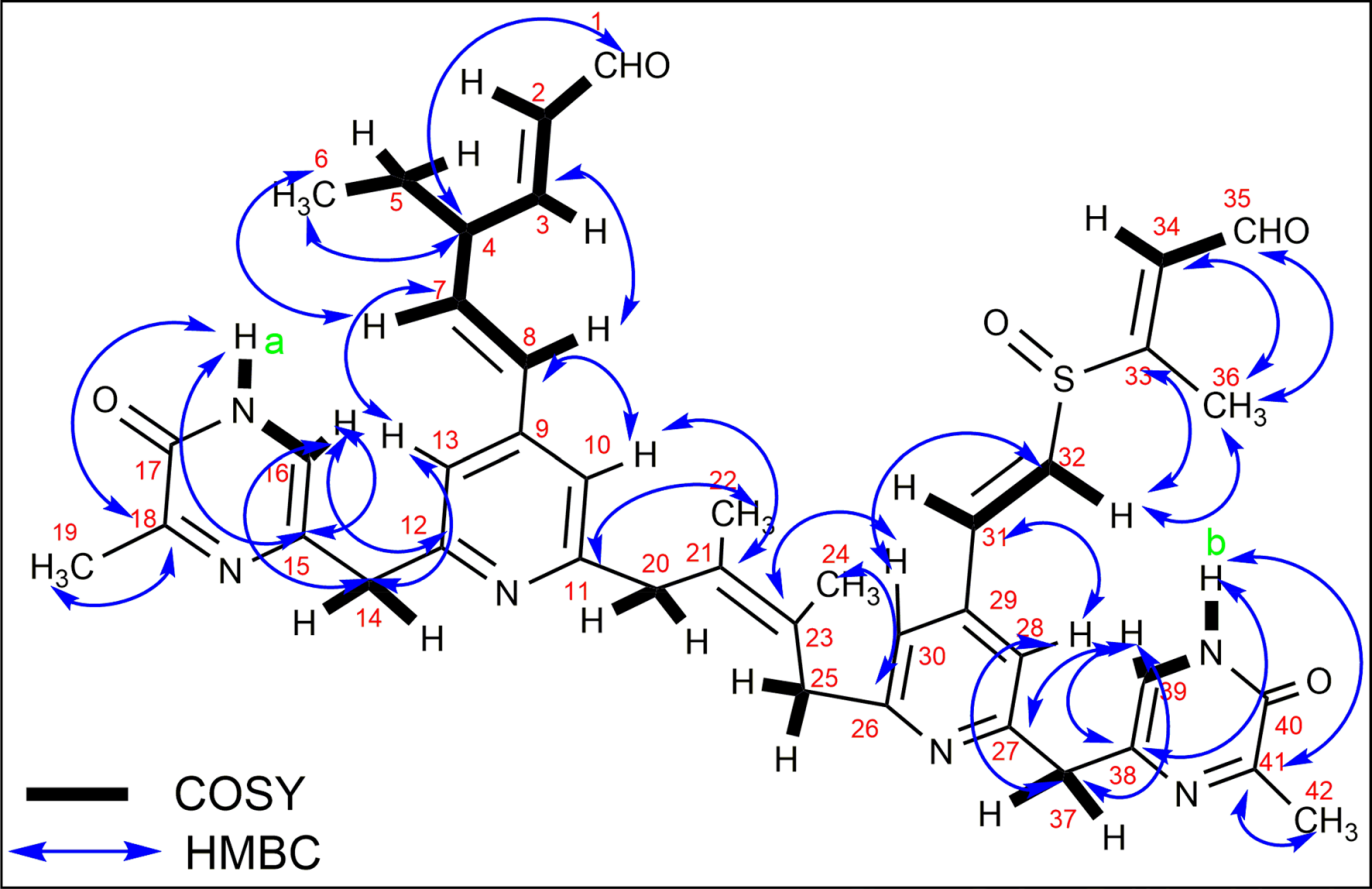
2D NMR (HMBC) correlation of pyrimidomycin.

Substructure A (Fig. 2) is the one hand of SM02. It contains a pyridine ring and a pyrimidine moiety attached with a methane (CH_2_) group. The ortho-coupled aromatic protons at δ 7.24 (H10, 13) and δ 6.14 (H8) were connected by the COSY spectrum (Fig. 3; Fig. S12).

An analysis of HMBC spectroscopic data provided further structural information on substructure A (Figs. 2, 3). H16 (at δ 7.75) showed a long-range correlation to δ 165.53(C 15) and δ 170.28 (C12). The cross peak from δ 7.21 (NH a) to δ 165.53 (C15) and δ 166.13 (C18), also from H_3_-19 (δ 2.03) to C18 supported the partial structure of the center ring. A long-range coupling from the proton signal of the pyridine unit at δ 7.24 (H13,10) to the methylene carbon δ 110.97 (C7) and δ 126.38 (C8) was observed. The presence of long-range coupling from the proton signal of olefinic proton δ 6.14 (H8) to methine carbon at δ 111.94 (C3) and proton δ 5.46 (H7) to methyl carbon at δ 12.66 (C6) has also been revealed. In HMBC spectra there is three-bond cross coupling between C4 (δ 51.82)-H_3_ 6 (δ 0.96) and four-bond cross coupling between C4 and aldehydic hydrogen H1 (δ 9.61), indicating the presence of substructure A (Fig. S13). This structural feature was further validated by HSQC NMR and NOESY NMR analysis (Fig. S14-17). In the NOSEY spectrum, H10 (7.24) proton interacts through space with H8 (6.14) and H22 (1.59) protons, further indicating the presence of structure A. Again, the space interaction between H8 (6.14)-H3 (5.75) and H4 (3.53) -H6 (0.96) determines the stereochemistry of C-4 carbon centre (Fig. S16, S17).

### Substructure B

Substructure B (Fig. 2) contained a pyridine ring and a pyrimidine moiety attached with a methane (CH_2_) group. The ortho coupled aromatic protons at δ 7.24 (H28, 30) and δ 6.32 (H31) were connected by the COSY spectrum (Fig. 3; Fig. S12). From the HMBC spectroscopic analysis the presence of pyrimidine moiety (in substructure B) attached with a pyridine via CH_2_ group has also been determined (same as substructure A) (Figs. 2, 3). A long-range coupling from the proton signal of the pyridine unit at δ 7.24 (H28,30) to the methylene carbon δ 130.53 (C31) and δ 133.57 (C32) was observed. The presence of long-range coupling from the proton signal of olefinic proton δ 6.63 (H32) to δ 143.43 (C33) and methyl carbon at δ 21.16 (C36) has also been revealed. The cross coupling of methyl group δ 1.38 (H_3_ 36) to C34 (δ 146.18) along with the aldehydic carbon C35 (δ 209.05) indicated the presence of substructure B.

In HMBC spectra, it has been observed that there are four-bond cross coupling between H10,30 (δ 7.24)-C21,23 (δ 122.03) and C11,26 (δ 170.28) -H22,24 (δ 1.59), which evident that the two substructures are connected through C21-C23 carbon as shown in Fig. 3. The proposed structure of SM02 is also established through HSQC spectrum (Fig S14, 15).

### Pyrimidomycin inhibits growth and biofilm formation of Gram-positive pathogen

**Figure 4:**
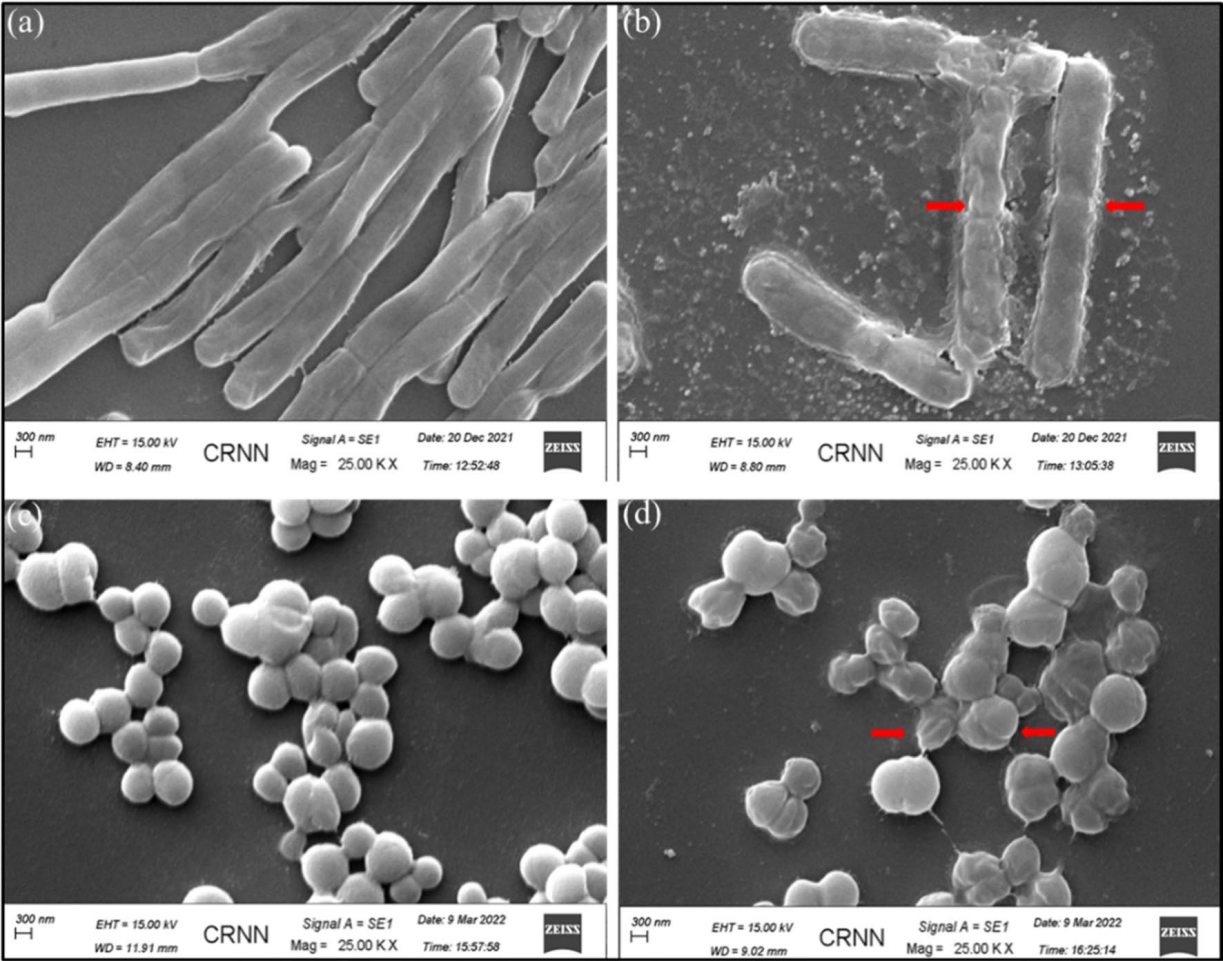
Pyrimidomycin induced membrane disruption of the test organisms *B. cereus* MTCC 1272 (**a**,**b**) and *S. aureus* MTCC 96 (**c**,**d**). (**a**,**c**) SEM images of untreated bacteria with smooth surface; (**b**,**d**) images showing cell compartment disruption (arrow-heads) visualized when the cells were treated with pyrimidomycin.

**Figure 5:**
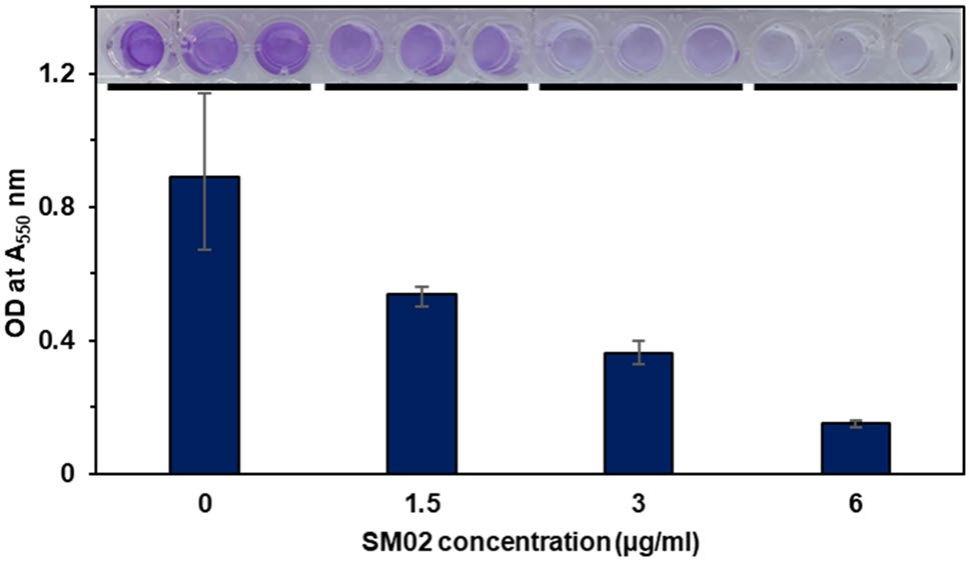
Antibiofilm properties of the compound pyrimidomycin. Increasing concentration leads to reduction of the biofilm formation.

The MIC and MBC values of the pyrimidomycin against various sensitive test organisms were determined (Table S3, Fig. S4). The MIC against the Gram-positive organisms like *S. aureus* MTCC 96, *S. pyogenes* MTCC 1928, *B. cereus* MTCC 1272, Methicillin-resistant *S. aureus* (MRSA), *M. smegmatis* mc^2^ 155 was found to be 12 μg/mL or 16.08 μM. However, we found that the MBC values are relatively higher and are more than 50 µg/mL. The cellular alteration was observed after the pyrimidomycin treatment to the test organisms. The cellular morphology of the untreated *B. cereus* MTCC 1272 and *S. aureus* MTCC 96 was intact (Fig. 4a,c) whereas the treated cells exhibited altered morphology (Fig. 4b,d), particularly the cellular membrane anticipated to be damaged severely. This data help to reveal the mode of action of the isolated pyrimidomycin. Further, the antibiofilm property of the pyrimidomycin was determined in sub-MIC value (1.56, 3 and 6 µg/mL) by crystal violet assay. In the case of positive control (untreated cells) the optical density was shown to be highest. The optical density was found to be decreased with the increasing concentration of pyrimidomycin indicating that the compound inhibits the biofilm formation by the test pathogen and thus could inhibit the colonization of the pathogen if applied (Fig. 5).

**Figure 6:**
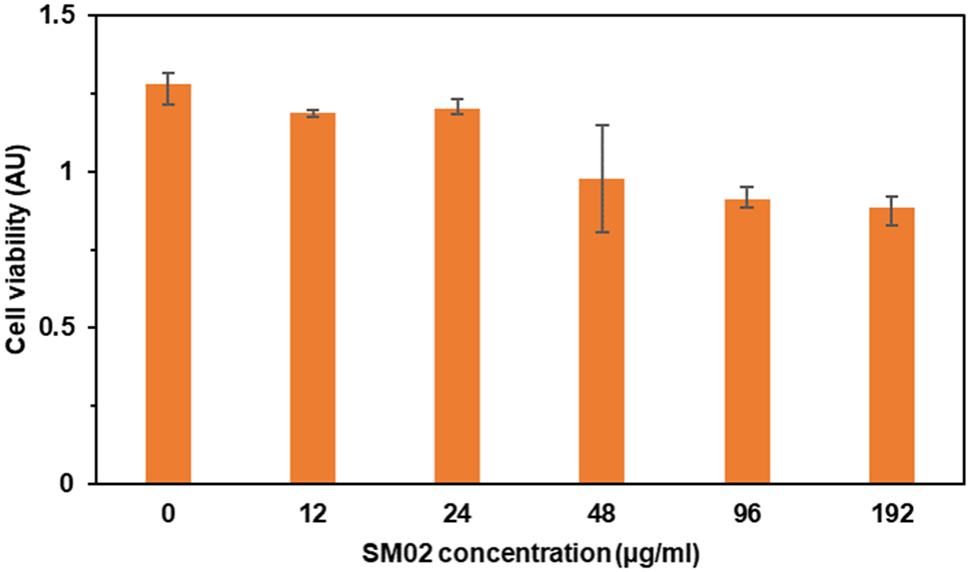
Cytotoxicity assay of pyrimidomycin against HuH-7 human cell line. Various concentration of pyrimidomycin was added in respective well having cells. Each concentration point was taken in triplicate.

The inhibitory effect of pyrimidomycin with different concentrations (12, 24, 48, 96 and 192 µg/ml) was also checked on human cell line (HuH-7) through MTT assay. The compound did not show any significant toxic effect on the cell line and can be predicted as non-toxic to humans (Fig. 6). However, the standard experiments on the mammalian models and the human subjects will confirm on its suitability for future use.

### Fractional inhibitory concentration index of pyrimidomycin

As we determine the MIC of pyrimidomycin is 12 µg/mL but when combined with other individual antibiotics, it shows its MIC much lower than its actual individual MIC value. The individual MIC value of ampicillin, chloramphenicol, vancomycin is 0.4 µg/mL, 4 µg/mL, 0.5 µg/mL, respectively. When 1/4 MIC of pyrimidomycin was combine with 1/4 MIC of ampicillin, chloramphenicol and vancomycin it showed complete growth inhibition of the test organism. FICI value of both shows equal to 0.5 which indicates pyrimidomycin has a synergistic effect (Table. 3).

**Table 3:** Synergistic relationship of pyrimidomycin with highly used popular antibiotics like ampicillin, chloramphenicol, vancomycin.

Test organism	Antibiotics	MIC of antibiotics in µg/mL (µM)	FICI*	Interpretation
*Staphylococcus aureus* MTCC 96	SM02	12 (16.08)	0.5	Synergy
	Ampicillin	0.4 (1.08)		
	SM02 + Ampicillin	3 (4.02)		
	Ampicillin + SM02	0.1 (0.27)		
	SM02	12 (16.08)	0.5	Synergy
	Chloramphenicol	4 (12.38)		
	SM02 + Chloramphenicol	3 (4.02)		
	Chloramphenicol + SM02	1 (3.09)		
	SM02	12 (16.08)	0.5	Synergy
	Vancomycin	1 (0.67)		
	SM02 + Vancomycin	3 (4.02)		
	Vancomycin + SM02	0.25 (0.17)		

*Fractional inhibitory concentration index (FICI) = (MIC of drug A in combination/MIC of drug A alone) + (MIC of drug B in combination/MIC of drug B alone). FICIs were interpreted as follows: ≤ 0.5 = synergy; 0.5–0.75 = partial synergy; 0.76–1.0 = additive effect; 1.0–4.0 = indifference; and > 4.0 = antagonism.

## Discussions

The studied *Streptomyces* strain was isolated from the soil sample collected from Manas National Park, Assam, India. The isolated strain PSAA01 has been identified as *Streptomyces* sp. which is capable of producing an antimicrobial compound named pyrimidomycin. The 16S rDNA sequence of the strain PSAA01 has been showing 99.72% similarity with *Streptomyces melanosporofaciens* DSM 40138. Small scale fermentation of the strain has been performed in different media with different temperatures, pH and incubation time to optimize the production of the pyrimidomycin. The used media contains different major nutritional sources such as carbon, nitrogen. ISP-2 contains yeast extract, malt extract, dextrose; oat meal is present in ISP-3; casein is contained by starch casein medium; TSB contains peptic digest of soyabean meal, dextrose; sodium propionate, sodium caseinate, L-asparagine and glycerol is present in AIA medium. We have found that ISP-2 medium is the optimum medium for the production of the antimicrobial by the strain PSAA01 at pH 7.0 upon 7 days of incubation. The structural elucidation has also been done which reveals that the molecule contains two substructures, substructure A and B. The molecular weight of this compound is 746 dalton. The molecule contains pyrimidine moiety and is yielded by *Streptomyces* sp., thus we named it pyrimidomycin.

Nowadays, bacterial multidrug resistance (MDR) is a challenging issue to the clinician as the control of the MDR pathogens becomes unsuccessful with most of the available antibiotics. Most of such pathogens are the causal agents for many life-threatening diseases in humans^9,39^. The isolated compound pyrimidomycin is specifically effective against Gram-positive organisms including the methicillin resistant *S. aureus* (MRSA) which shows their resistance property against typical beta-lactam drugs like methicillin, amoxicillin, penicillin, nafcillin, oxacillin and cephalosporin^40^. The minimum inhibitory concentration of the pyrimidomycin is 12 µg/mL against *S. aureus* MTCC 96*, S. pyogenes* MTCC 1928*, B. cereus* MTCC 1272*,* Methicillin resistant *S. aureus, M. smegmatis* mc^2^ 155 and 24 µg/mL for *B. subtilis* MTCC 441 (Table S3)*.* The antibiofilm activity of the pyrimidomycin has been recorded at the sub-MIC values. The SEM assay has been showing the cell wall and cell membrane rupture after the treatment of *B. subtilis* MTCC 441 or *S. aureus* MTCC 96 with the MIC value (12 µg/mL). The pyrimidomycin does not affect tested human HuH-7 cell lines up to the concentration of 48 µg/ml concentration. From these experiments it can be concluded that the identified strain is a novel antibiotic producer. The pyrimidomycin has been successfully characterized and the presumable mode of action is also indicated in this study. The inhibitory effect of the compound on drug-resistance strain is a promising outcome. Furthermore, as pyrimidomycin shows a synergistic relationship with very common existing antibiotics like ampicillin, vancomycin and chloramphenicol, it might be a choice of antimicrobials in the future for its combinatorial use.

## Supplementary Information

Supplementary Information.

## Data Availability

The bacterial culture has been submitted to MTCC, India with the accession number MTCC 13157. The 16S rDNA sequence of the strain was deposited in NCBI and the accession number is MT829328. All the data associated with the research are available as supplementary files.
