# Supplementary material for: An antibacterial compound pyrimidomycin produced by Streptomyces sp. PSAA01 isolated from soil of Eastern Himalayan foothill
Source: Sci Rep. 2022 Jun 17;12:10176. doi: 10.1038/s41598-022-14549-4 (PMC9206078; doi:10.1038/s41598-022-14549-4)
Supplement: Supplementary file 1 — Supplementary Information. [file 41598_2022_14549_MOESM1_ESM.pdf]

Title: An antibacterial compound pyrimidomycin produced by *Streptomyces* sp. PSAA01 isolated from soil of Eastern Himalayan foot-hill.

Author: Prasenjit Das[#1], Shampa Kundu[#2], Pulak Kumar Maiti[1], Saurodeep Mandal[2], Prithidipa Sahoo[2*], Sukhendu Mandal[1*]

Affiliations:

[1]Laboratory of Molecular Bacteriology, Department of Microbiology, University of Calcutta, 35, Ballygunge Circular Road, Kolkata, 700019, India

[2]The Molecular Recognition Laboratory, Department of Chemistry, Visva-Bharati University, Siksha Bhavana, Santiniketan, Birbhum, West Bengal-731235, India

[#] These two authors contributed equally

* To whom correspondence should be addressed: sukhendu1@hotmail.com

Corresponding Author: Sukhendu Mandal

Address: Laboratory of Molecular Bacteriology, Department of Microbiology, University of Calcutta, 35, Ballygunge Circular Road, Kolkata, 700019, India.

Email: sukhendu1@hotmail.com

Co-corresponding Author: Prithidipa Sahoo

Address: Molecular Recognition Laboratory, Department of Chemistry, Visva-Bharati University, Siksha Bhavana, Santiniketan, Birbhum, West Bengal-731235, India.

Email: prithidipa@hotmail.com

Table S1. Optimization of the production of SM02 in different media.

Name of media	Wet-weight (gm/50 ml)	Zone of inhibition (mm) (*S. aureus* MTCC 96)
**ISP2**	2.77	25
**TSB**	1.54	-
**ISP3**	4.16	15
**Starch casein**	1.2	-
**AIA**	1.4	-

Table S2. IR values and corresponding group of SM02

Absorption (cm^{-1})	Appearance	Group	Compound class
3354	medium	N-H stretching	Amide
2934	medium	C-H stretching	Alkane
2832	medium	C-H stretching	Aldehyde
1710	medium	C=O stretching	Aldehyde
1661	medium	C=O stretching	Amide
1456	medium	C-H bending	Alkane (methyl group)
1380	weak	C-H bending	Aldehyde
1027	strong	S=O stretching	Sulfoxide
958	medium	C=C bending	Alkene (trans disubstituted)
744	weak	C=C bending	Alkene (trisubstituted)

Table S3. Efficacy of pyrimidomycin against tested pathogens.

**Test organisms**	**MIC (µM)**	**MBC (µM)**
S. aureus MTCC 96	16.08	>50
S. epidermidis MTCC 3086	16.08	>50
B. cereus MTCC 1272	16.08	>50
B. subtilis MTCC 441	32.16	>50
S. pyogenes MTCC 1928	16.08	>50
Methicillin resistant *S aureus*	16.08	>50
M. smegmatis mc^2 155	16.08	>50

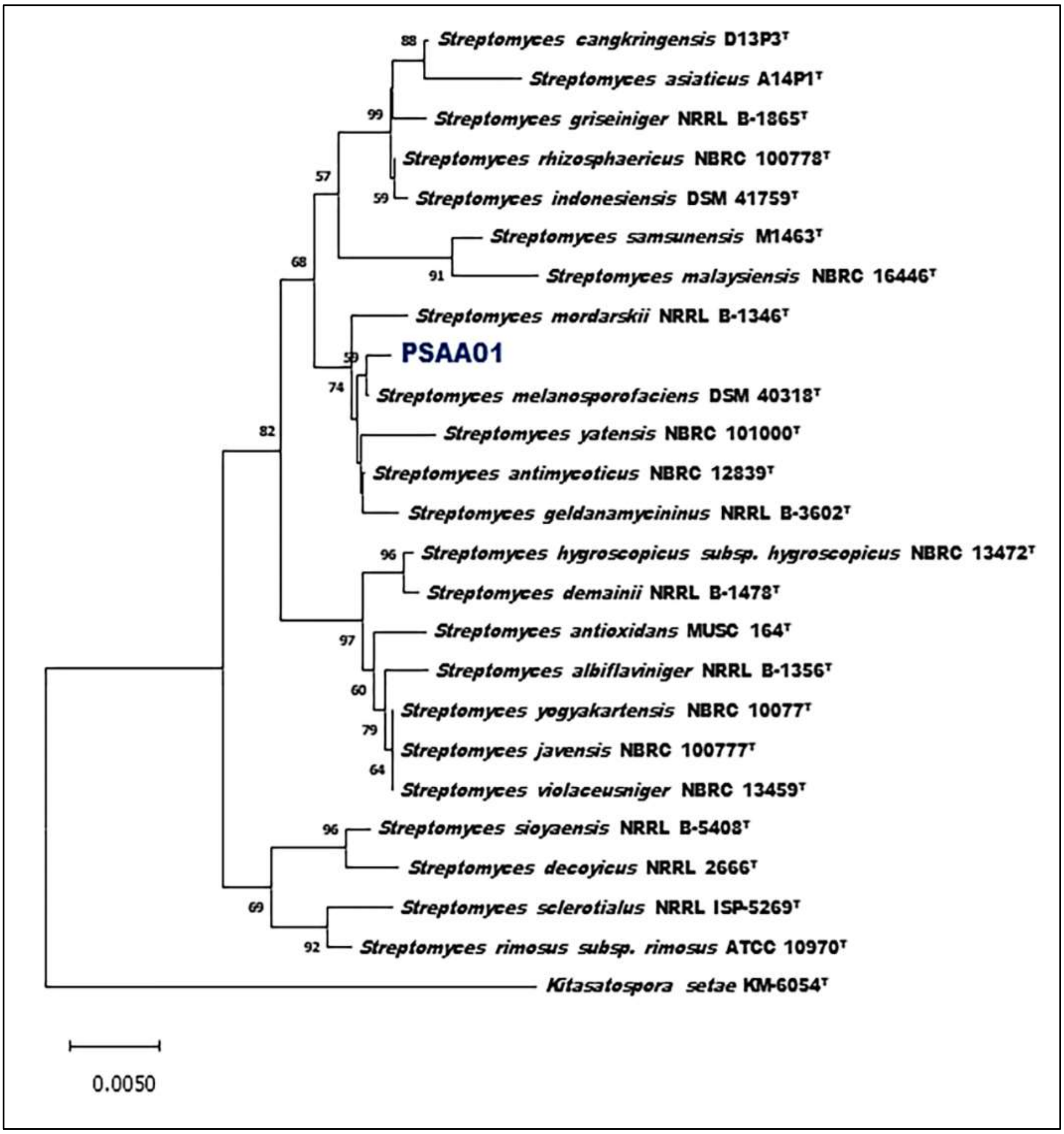

Fig. S1. Showing the Neighbour joining (NJ) phylogenetic tree of PSAA01 and its closest relatives on the basis of 16S rDNA. The bootstrap values (>50) are represented at the nodes.

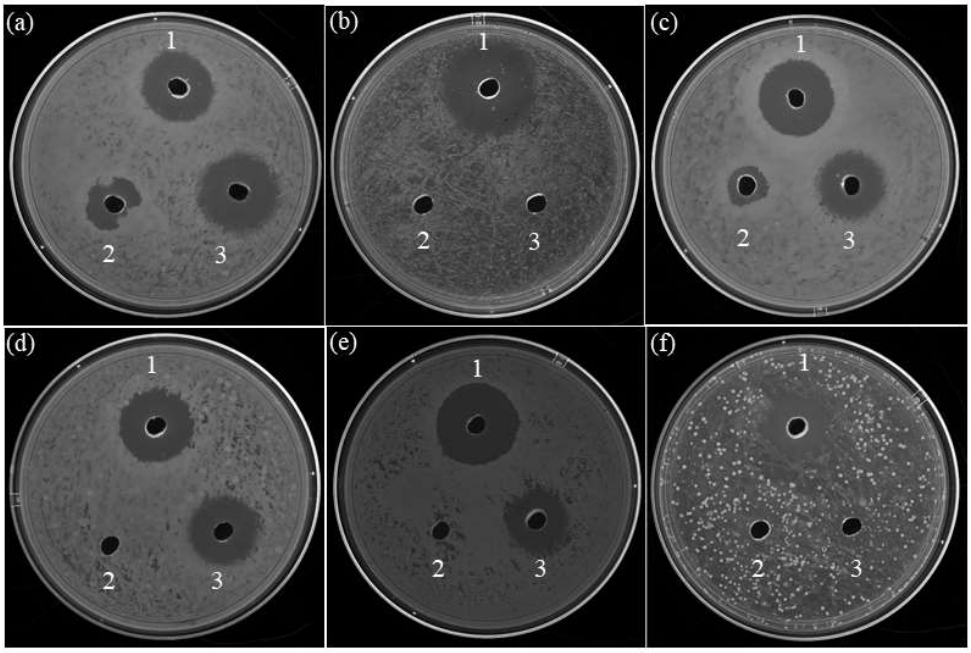

Fig. S2. Antimicrobial efficacy of crude extract having SM02. The efficacies were detected in Muller-Hinton media by zone of inhibition against different organisms using cell free extract consisting of SM02. (a) = *S. aureus* MTCC 96, (b) = *S. aureus* MRSA, (c) = *B. cereus* MTCC 1272, (d) = *B. subtilis* MTCC 441, (e) = *S. pyogenes* MTCC 1928, (f) = *M. smegmatis* mc^2 155; and1 = SM02 crude, 2 = ampicillin (10 µg), and 3 = chloramphenicol (4 µg)

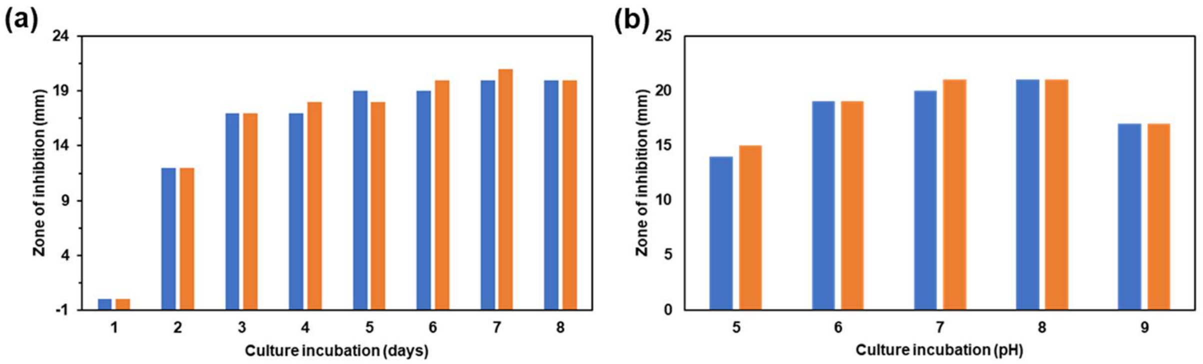

Fig. S3. Optimization of the production of SM02 from PSAA01 in culture incubation time (a) and media pH (b).

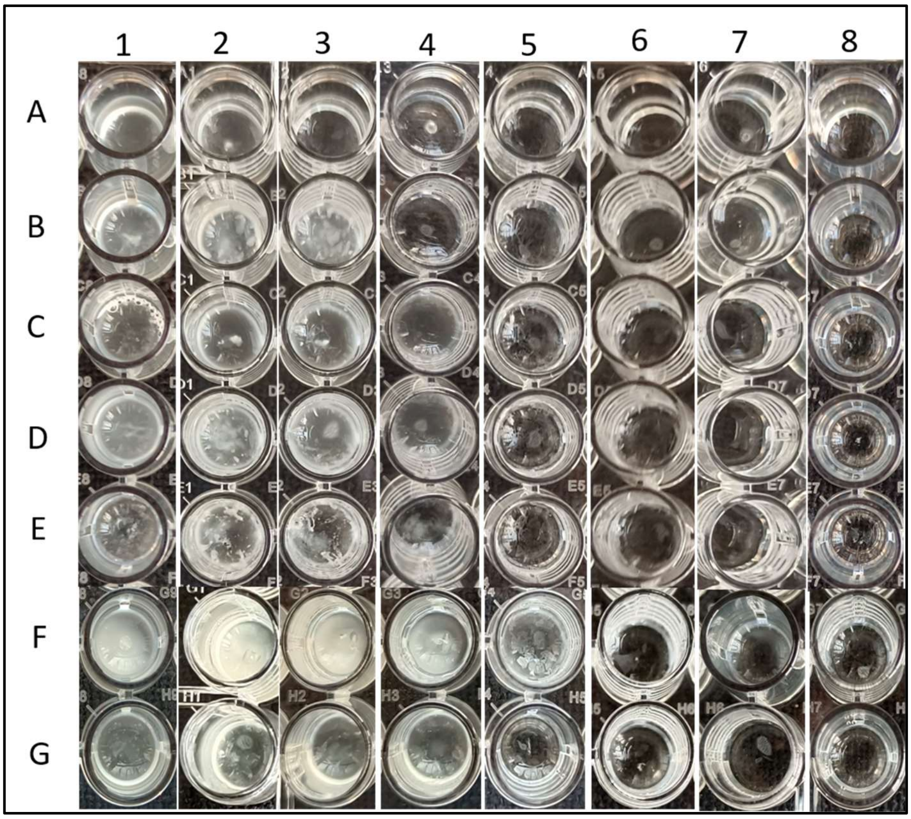

Fig. S4. MIC of SM02 compound against different test organisms. A= *S. aureus* MRSA, B= *B. subtilis* MTCC 441, C= *S. epidermidis* MTCC 3086, D= *S. pyogenes* MTCC 1928, E= *S. aureus* MTCC 96, F= *M smegmatis* mc^2155, G= *B. cereus* MTCC 1272*,* different concentrations are 1= control without antibiotic 2= 0.375 µg, 3=0.75µg 4=1.5 µg, 5=3 µg, 6=6 µg, 7=12 µg, 8=24 µg.

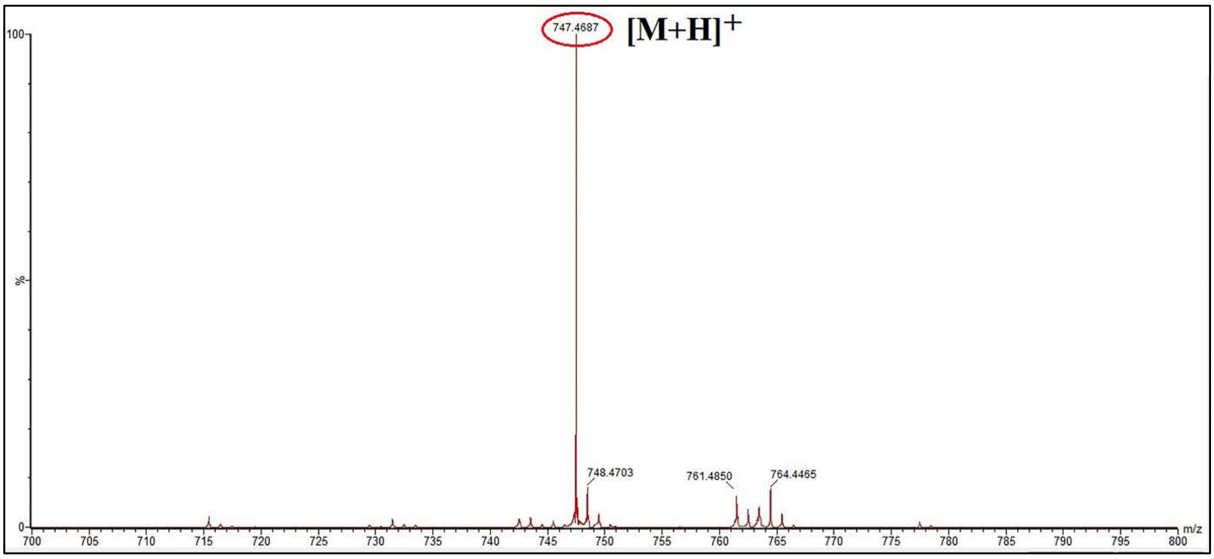

Fig. S5. HRMS spectra of SM02

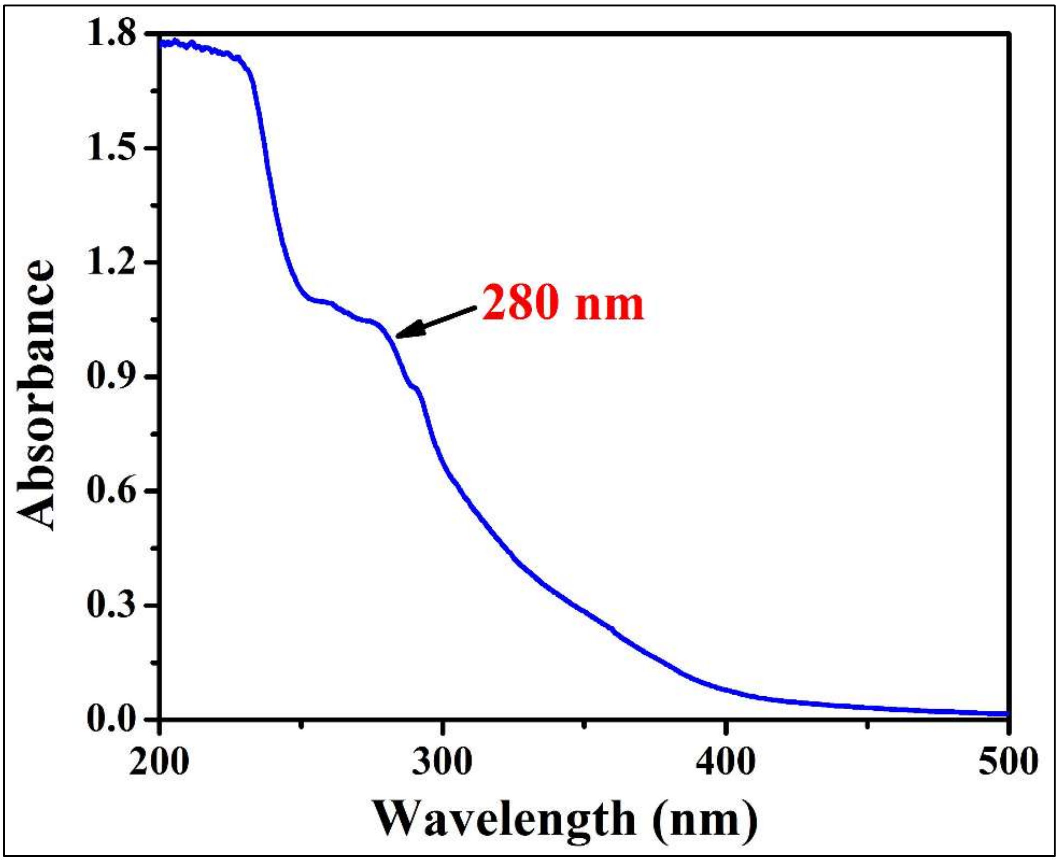

Fig. S6. Absorbance plot of SM02

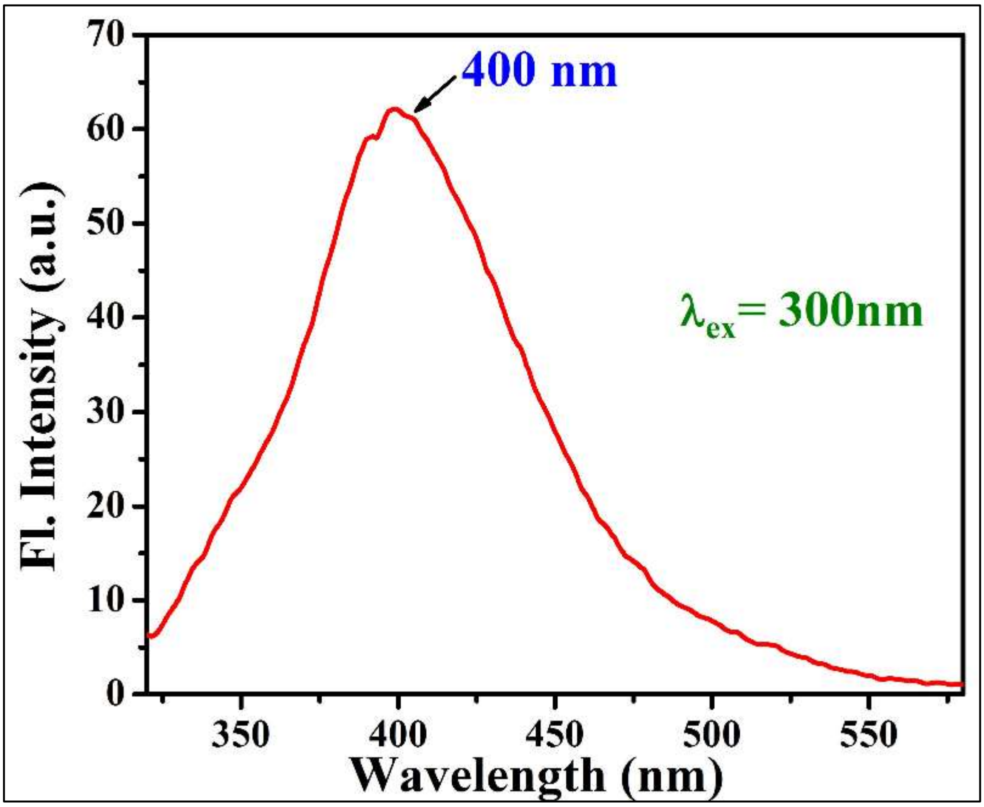

Fig. S7. Fluorescence plot of SM02

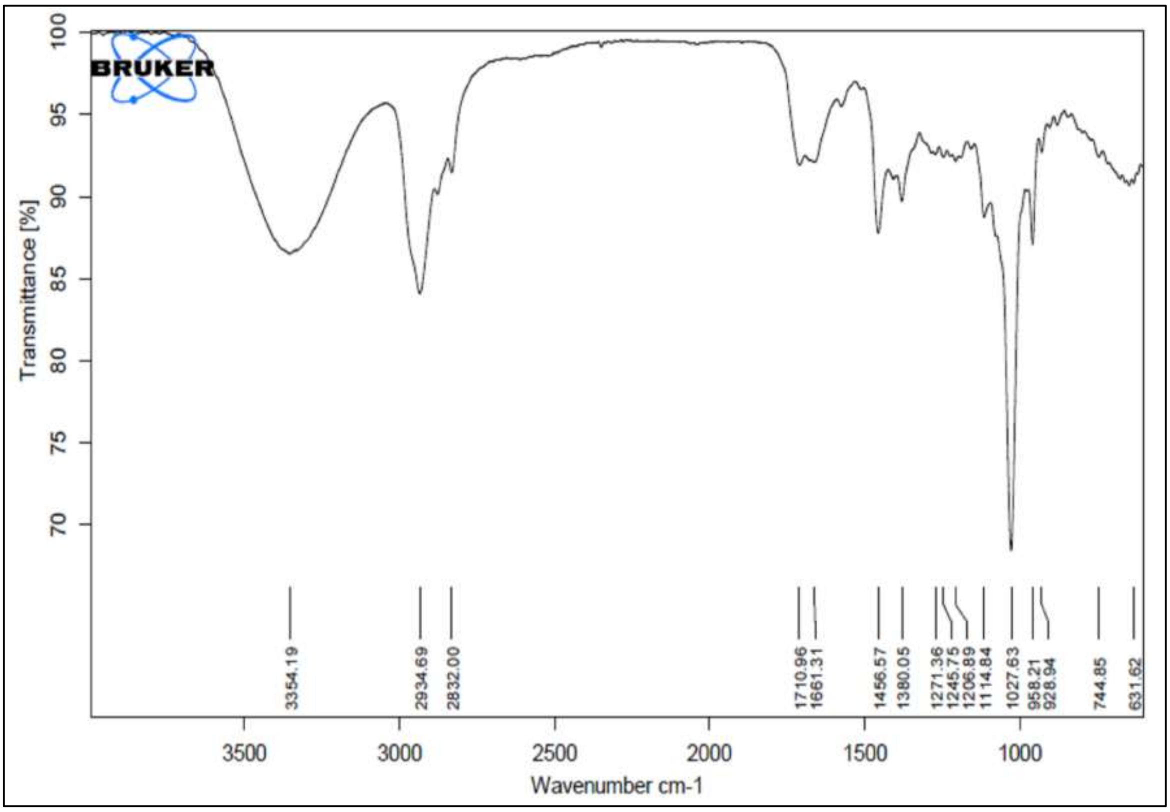

Fig. S8. FTIR spectra of SM02

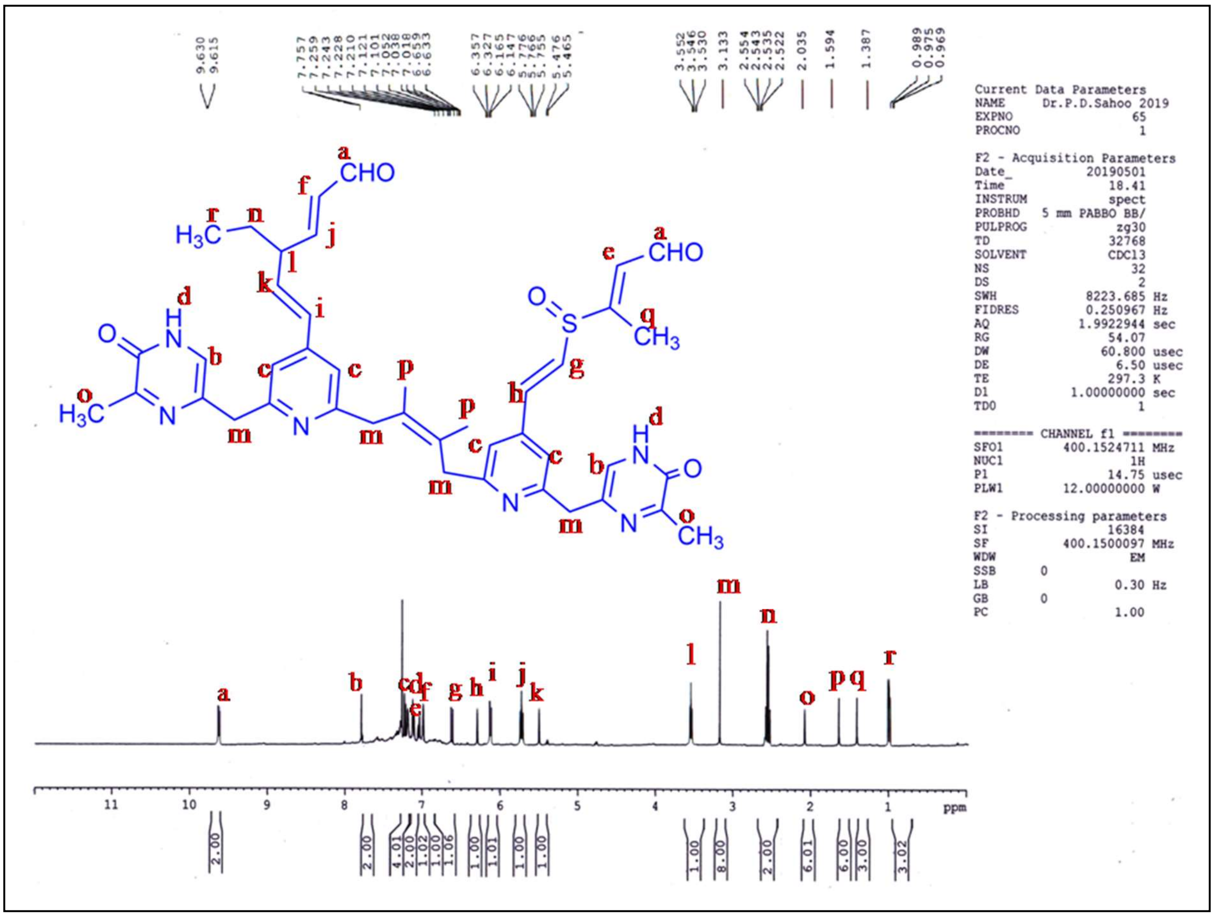

Fig. S9. ^{1}H NMR of SM02 in $CDCl_3$ (400 MHz)

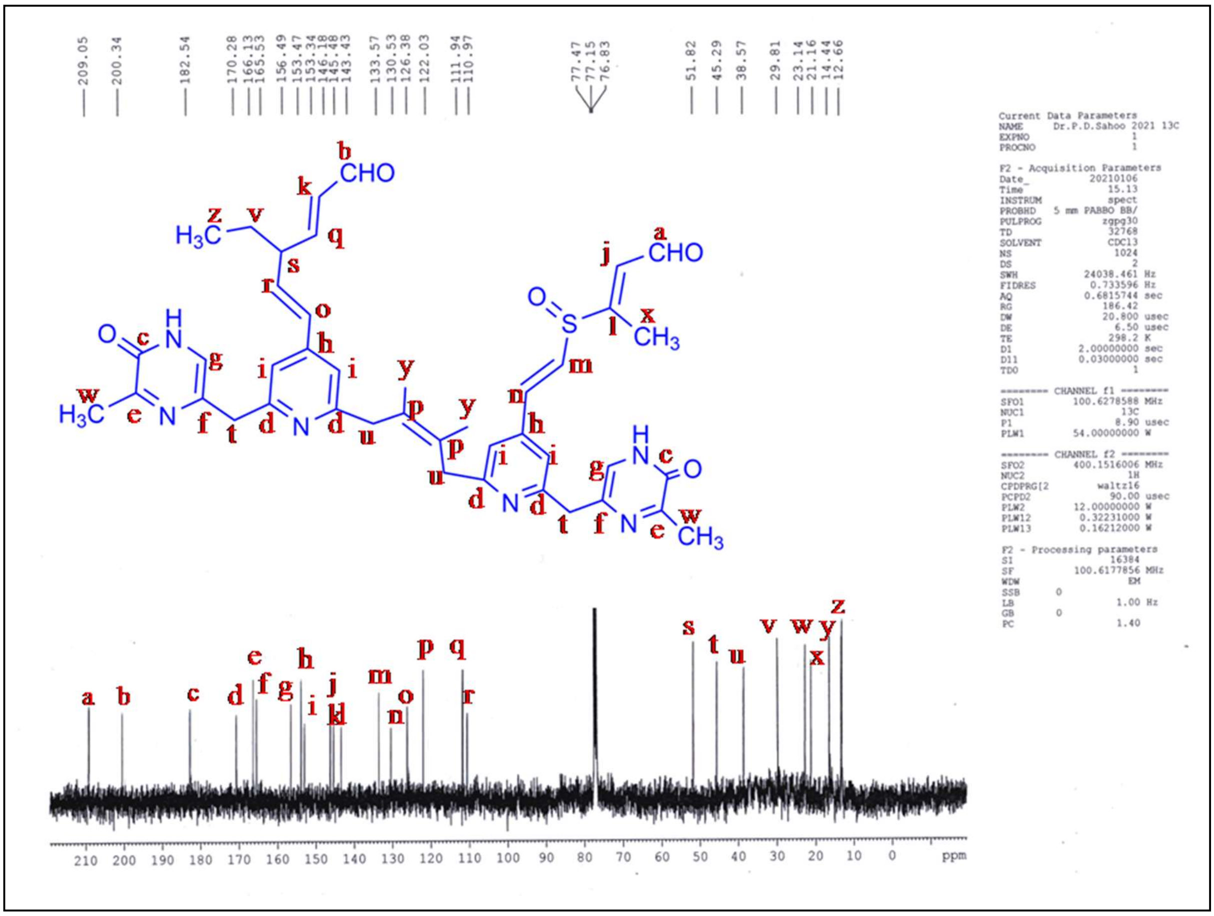

Fig. S10. ^{13}C NMR of SM02 in $CDCl_3$ (100 MHz)

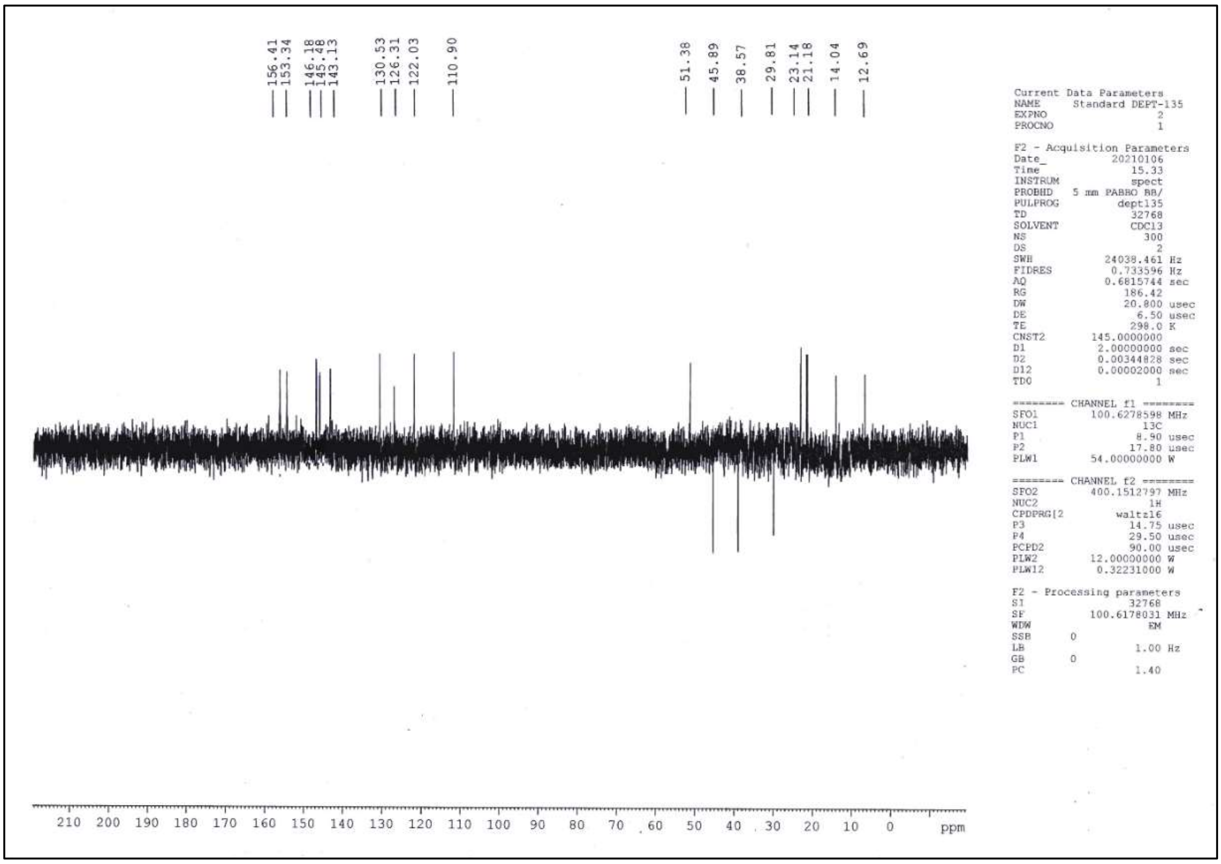

Fig. S11. ^{13}C-DEPT-135 NMR of SM02 in $CDCl_3$

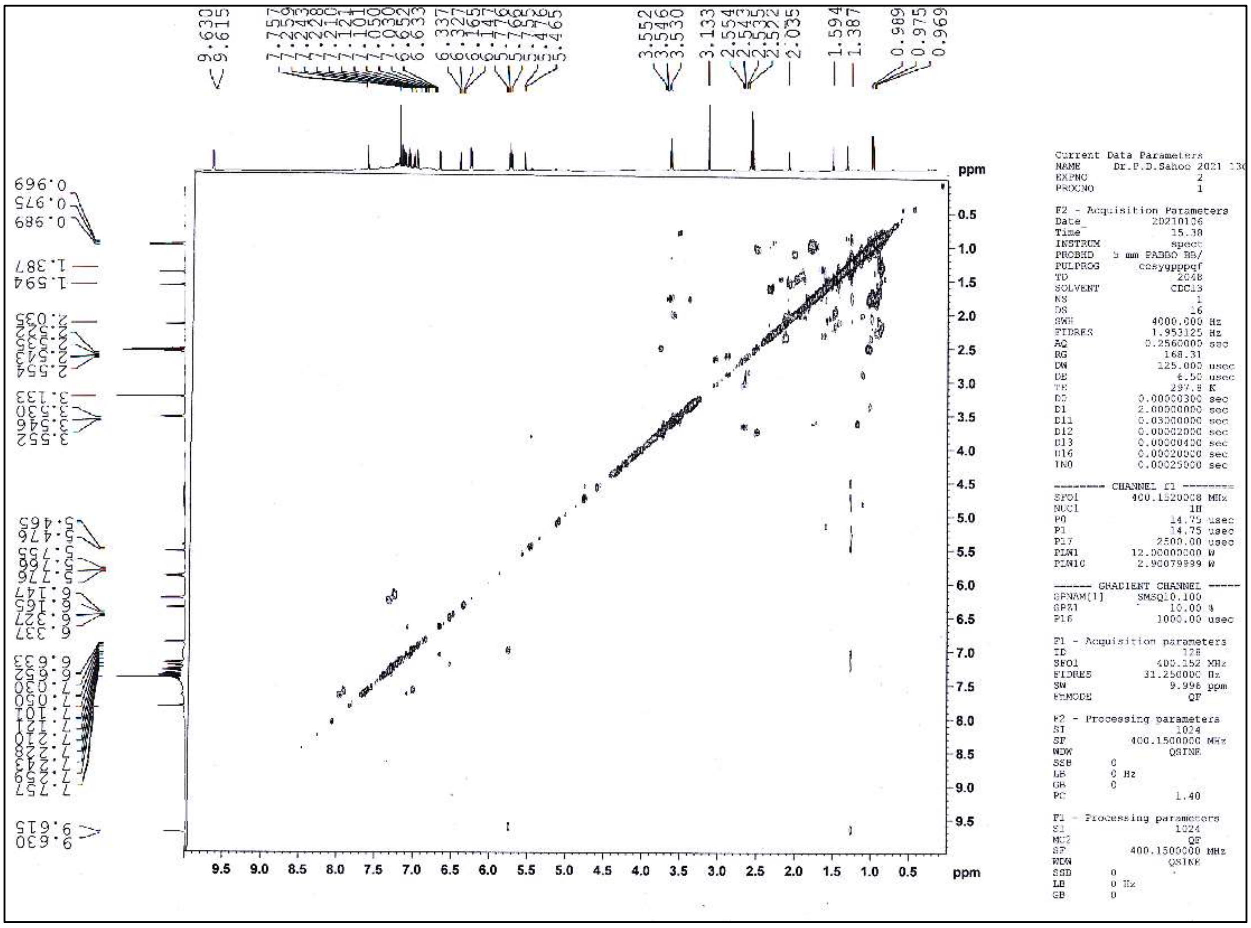

Fig. S12. COSY NMR of SM02 in $CDCl_3$

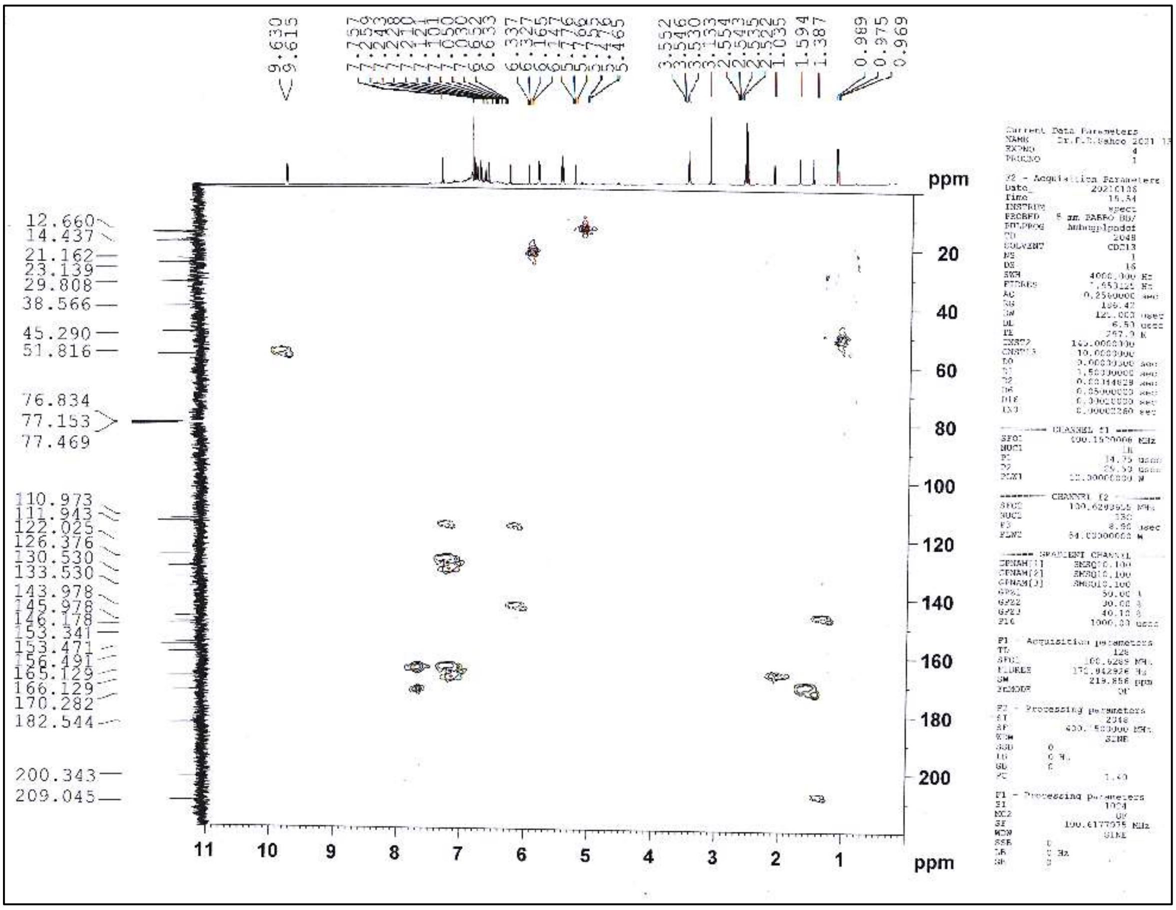

Fig. S13. HMBC NMR of SM02 in $CDCl_3$

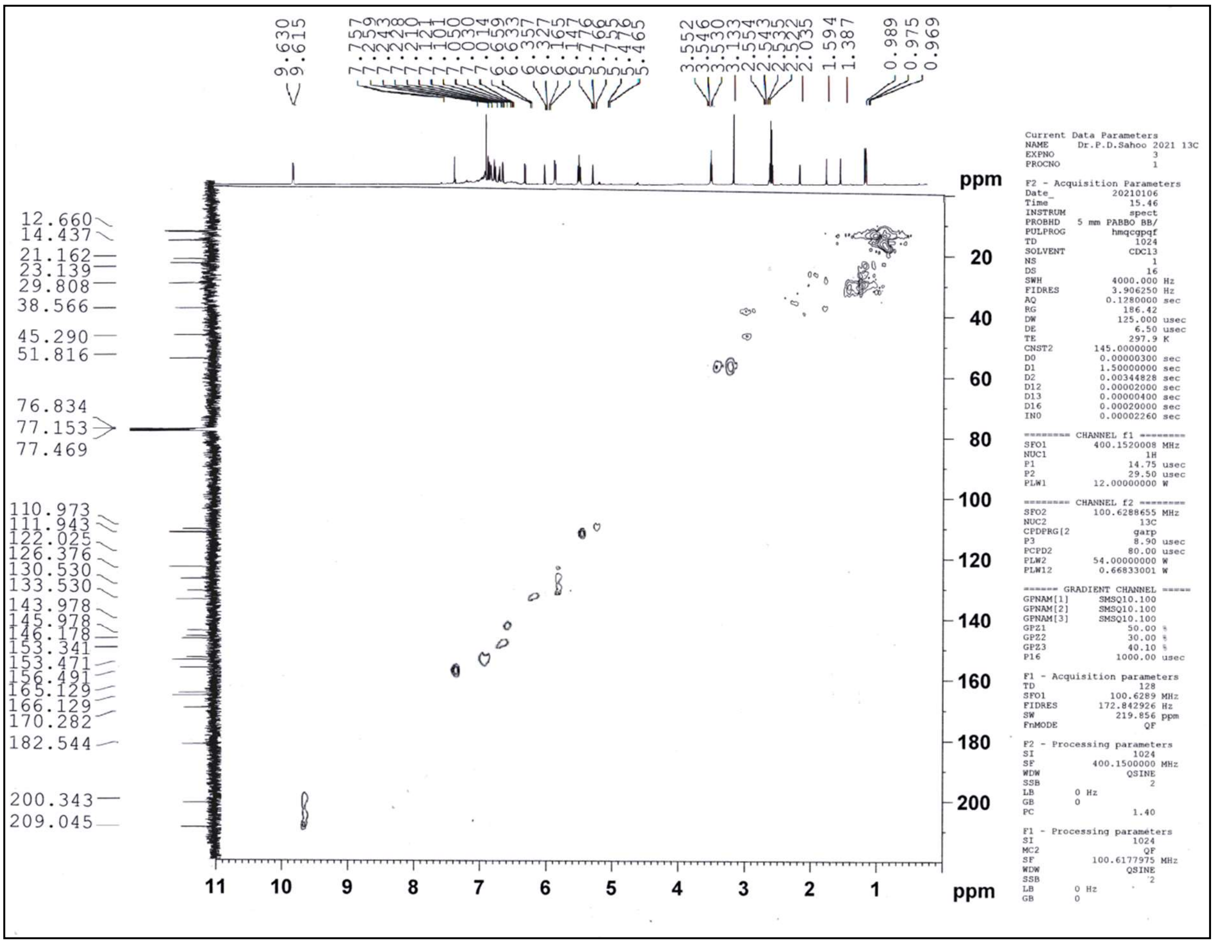

Fig. S14. HSQC NMR of SM02 in $CDCl_3$

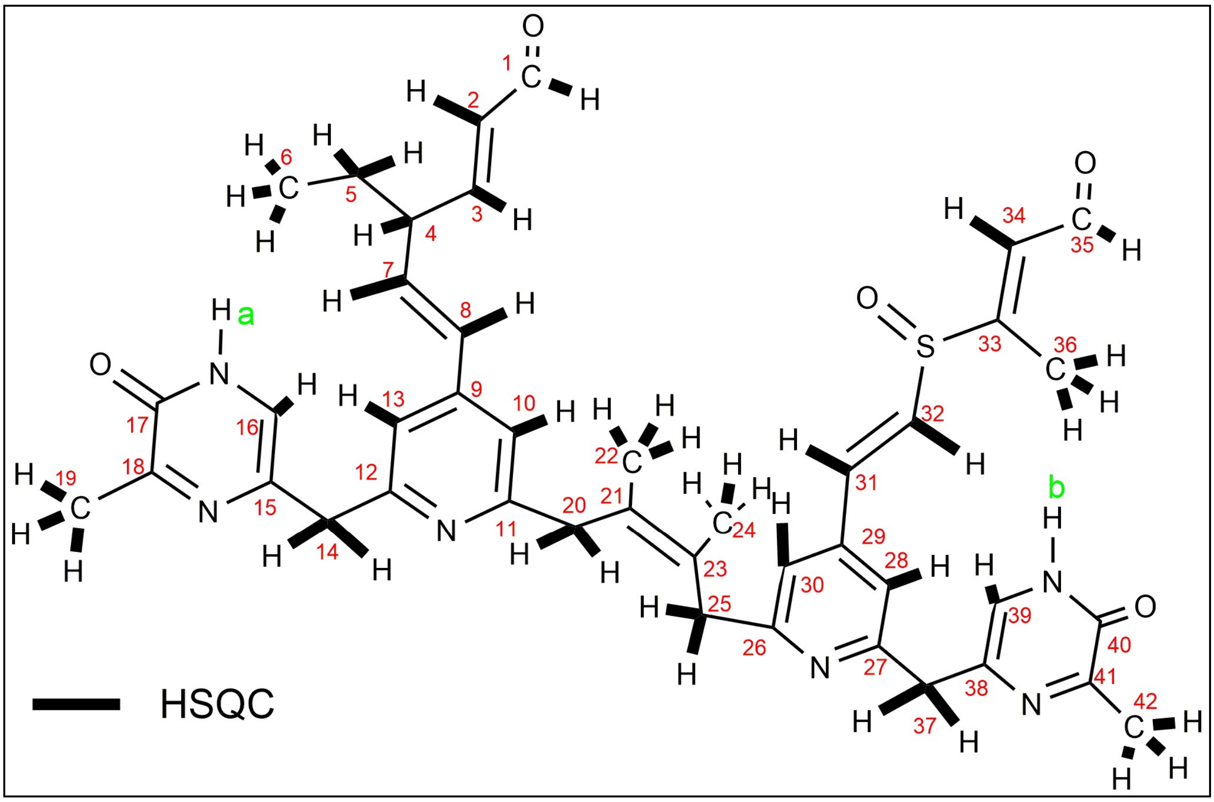

Fig. S15. 2D NMR (HSQC) correlation of SM02

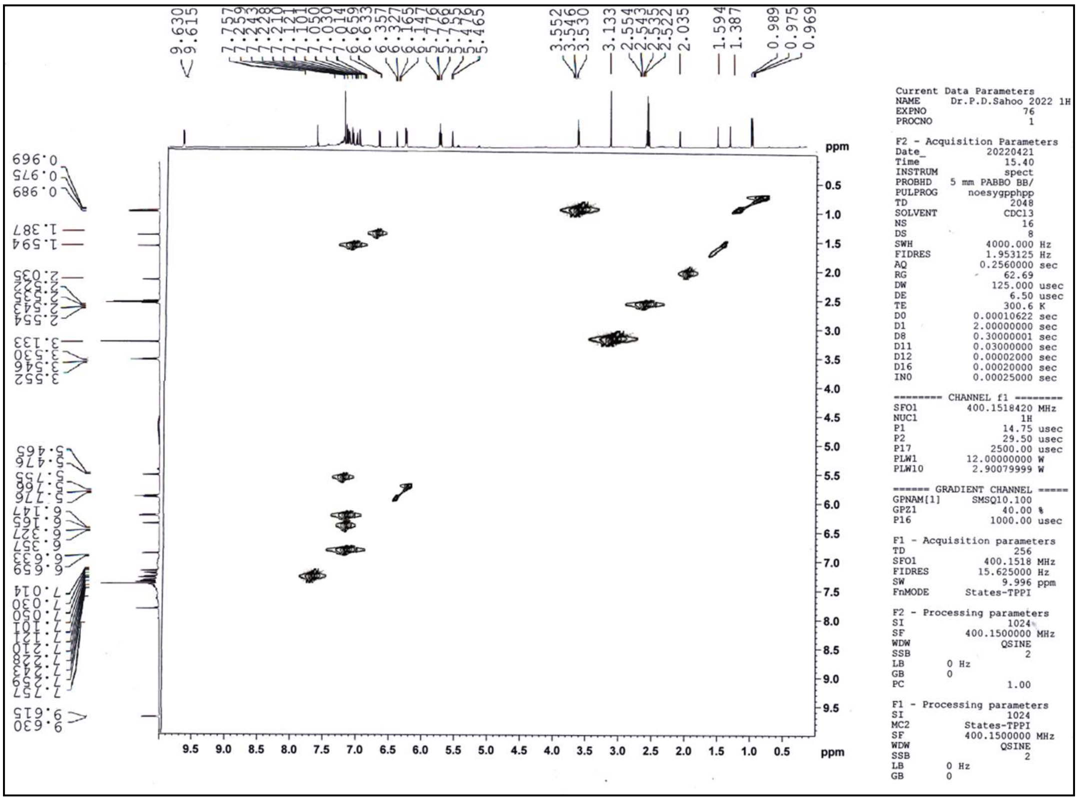

Fig. S16. NOSEY NMR of SM02 in $CDCl_3$

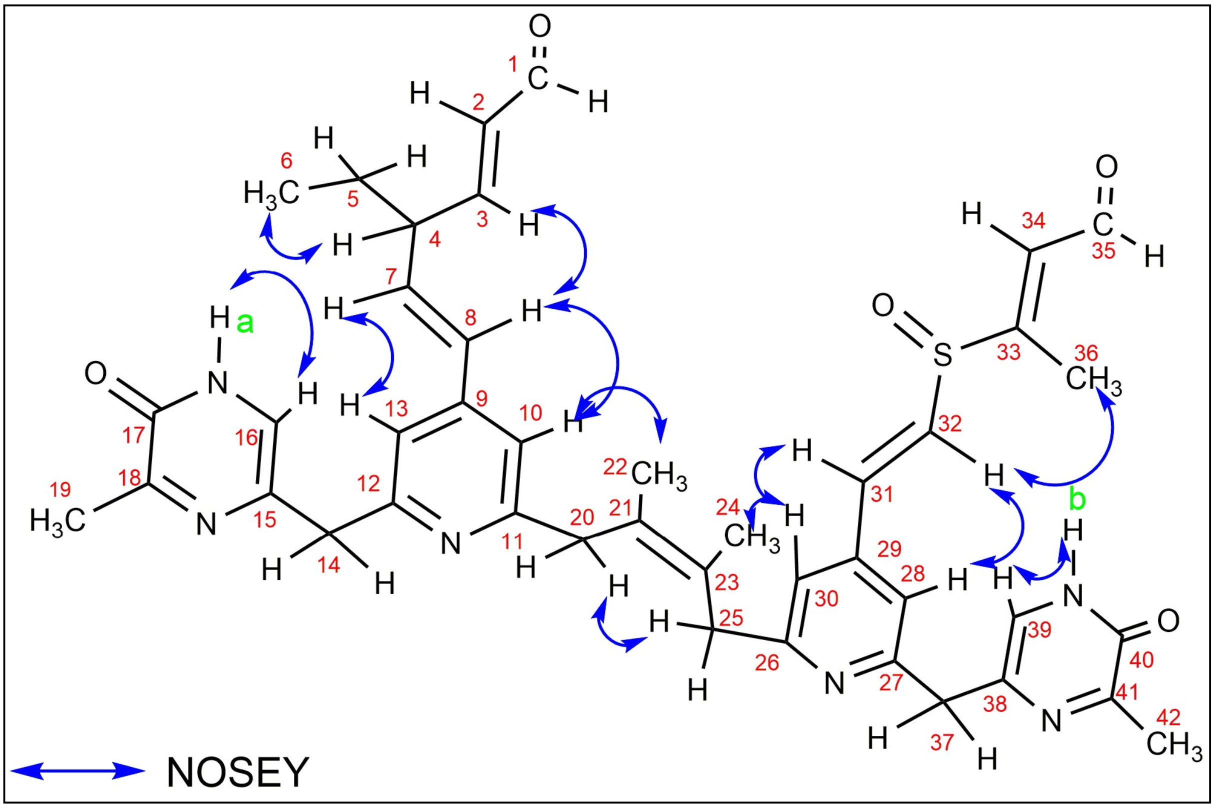

Fig. S17. 2D NMR (NOSEY) correlation of SM02
